# FKG-MM: A multi-modal fuzzy knowledge graph with data integration in healthcare

**DOI:** 10.1371/journal.pone.0339864

**Published:** 2026-01-02

**Authors:** Nguyen Hong Tan, Tran Manh Tuan, Pham Minh Chuan, Nguyen Duc Hoang, Le Quang Thanh, Le Hoang Son

**Affiliations:** 1 Graduate University of Science and Technology, Academy of Science and Technology, Hanoi, Vietnam; 2 Faculty of Information Technology, Thai Nguyen University of Information and Communication Technology (ICTU), Thai Nguyen, Vietnam; 3 Institute of Information Technology, Vietnam Academy of Science and Technology, Hanoi, Vietnam; 4 Artificial Intelligence Research Center, VNU Information Technology Institute, Vietnam National University, Hanoi, Vietnam; 5 Faculty of Computer Science and Engineering, Thuyloi University, Hanoi, Vietnam; 6 Faculty of Information Technology, Hung Yen University of Technology and Education, Hung Yen, Vietnam; 7 School of Dentistry, Hanoi Medical University, Hanoi, Vietnam; Minnan Normal University, CHINA

## Abstract

Artificial Intelligence (AI) has been dramatically applied to healthcare in various tasks to support clinicians in disease diagnosis and prognosis. It has been known that accurate diagnosis must be drawn from multiple evidence, namely clinical records, X-Ray images, IoT data, etc called the multi-modal data. Despite the existence of various approaches for multi-modal medical data fusion, the development of comprehensive systems capable of integrating data from multiple sources and modalities remains a considerable challenge. Besides, many machine learning models face difficulties in representation and computation due to the uncertainty and diversity of medical data. This study proposes a novel multi-modal fuzzy knowledge graph framework, called FKG-MM, which integrates multi-modal medical data from multiple sources, offering enhanced computational performance compared to unimodal data. In addition, the FKG-MM framework is based on the fuzzy knowledge graph model, one of the models that represent and compute effectively with medical data in tabular form. Through some experiment scenarios utilizing the well-known BRSET dataset on multi-modal diabetic retinopathy, it has been experimentally validated that the feature selection method, when combining image features with tabular medical data features, gives the highest reliability results among 5 methods including Feature Selection Method, Tensor Product, Hadamard Product, Filter Selection, and Wrapper Selection. In addition, the experiment also confirms that the accuracy of FKG-MM increases by 12–14% when combining image data with tabular medical data than the related methods diagnosing only on tabular data.

## Introduction

Recent advances in Artificial Intelligence (AI) technologies have significantly transformed numerous domains, ranging from transportation and finance to healthcare and education. The utilization of AI methods in healthcare and medicine, particularly Machine Learning (ML) and Deep Learning (DL), has become popular and has created many different models for diagnosis, prognosis, and prediction of several diseases. The predominant dependence on unimodal data sources, such as X-ray images, intraoral photos, computed tomography (CT) scans, magnetic resonance imaging (MRI), endoscopy images, etc. poses distinct challenges for contemporary healthcare applications. These models often lack the integration of critical complementary data sources and multiple modalities, thereby constraining their ability to deliver in-depth insights [1]. However, despite their effectiveness, most current methods practice of combining diverse sources of information to enhance decision-making [2,3].

As many diverse data source in healthcare, including medical imaging and electronic health records, become increasingly accessible, the demand for effective integration and fusion of the multi-modal information are generally based on data from only one modality, which restricts their ability to accurately mirror the clinical has grown, aiming to enable more comprehensive analysis and informed clinical decision-making [4]. The integration of data from diverse modalities, commonly referred to as **multi-modal data fusion**, has significantly influenced advancements in the healthcare domain [5]. The fusion of multi-modal data within healthcare fields is consistent with the foundational concepts of predictive, preventive, and personalized medicine (3 PM), as it leverages heterogeneous data sources to enhance clinical decision-making [6]. This integrative methodology supports the development of predictive models, facilitates early intervention strategies, and promotes personalized treatment plans, ultimately enhancing patient outcomes and optimizing the efficiency of healthcare delivery.

In particular, Dongwei Xie et al. [7] introduced a multi-modal fusion framework utilizing a late fusion method to merge RGB and skeletal features, thereby improving the model’s capacity to effectively capture both spatial and temporal dependencies. A model MDF-Net proposed by Hsieh et al. [8] derived from and extended upon the Mask R-CNN framework is designed to simultaneously integrate clinical and chest X-ray images data, enabling more accurate identification of abnormal regions within chest radiographs. The adoption of integrating multi-modal data presents a promising approach, facilitating the development of AI-driven healthcare systems that offer more refined diagnostic assessment, accurate prognostic predictions, and tailored therapeutic interventions [4].

Research in the field of multi-modal medical diagnosis remains in its early developmental phase, with ongoing efforts focused on formulating novel methodologies to effectively process and analyze multi-modal medical data [9]. For example, COVID-19 has manifested as a highly lethal global pandemic, leading to the death of more than three million individuals across the world [10]. In response, a multi-modal diagnostic framework named Ai-CovScan was introduced to detect COVID-19 by integrating data from chest X-ray imaging, respiratory sounds, and rapid antigen test outcomes. This framework demonstrated a preliminary accuracy of 80% in analyzing breathing sound and achieved a COVID-19 detection accuracy of 99.66% on the chest X-ray image dataset [10]. AutoPrognosis-M presents a multi-modal methodology that facilitates the integration of structured clinical (tabular) data and medical imaging through automated ML techniques [2]. Similarly, Silva and Rohr [11] developed MultiSurv, a multi-modal approach designed for the diagnosis of over 30 cancer types. Furthermore, a study by Lu [12] introduced a multi-modal DL model that leverages multidimensional and multi-level temporal data to predict multi-drug resistance in patients with pulmonary tuberculosis.

Despite these advancements, prior studies have predominantly concentrated on the fusion of metadata and imaging features, often overlooking the exploration of the intrinsic relationship between these two modalities. Imaging and tabular data have been identified as the most frequently utilized modalities [13]. Imaging modalities serve a critical function in the diagnosis and monitoring of numerous medical conditions, benefiting from their integration into clinical workflows, standardized protocols, and the availability of specialized expertise for interpretation. Tabular data, representing structured clinical information, is equally vital for the holistic evaluation and management of patients, serving as a cornerstone for the implementation of personalized medicine [13]. Metadata offers complementary contextual information that can enhance the interpretation of imaging data, while image features inherently contain distinctive visual cues that may inform a more nuanced understanding of metadata [14]. Therefore, the integration of these modalities holds the potential to more effectively reveal features critical for accurate disease detection and classification.

Multi-modal data fusion enhances the precision and depth of clinical decision support systems by enabling a more holistic understanding of patient information in medical data analysis [15]. These graphs serve as essential frameworks for knowledge representation and reasoning, facilitating more informed clinical inferences [15]. **Knowledge graphs (KGs)** structurally encode factual information through structured triples, composing of head and tail entities connected by a binary relation [16]. With the rapid evolution of KG technologies, they have been widely adopted in various applications, including semantic search [17], recommender systems [18], and question-answering tasks [19,20]. As a fundamental component of AI, KGs offer a powerful framework for knowledge representation; however, they often struggle to address ambiguities inherent in fuzzy semantic contexts [21]. To address this limitation, fuzzy knowledge graphs (FKGs) have been proposed as semantic networks that not only capture entity relationships but also provide a formal mechanism for representing real-world concepts and their relationships [22]. Despite their potential, existing FKG research has largely been confined to experiments using unimodal datasets, typically based on symptom data derived from test indicators, without evaluating performance on multi-modal datasets originating from many diverse sources [23–25]. Furthermore, these FKG studies have yet to assess the influence of input features on predictive outcomes. Identifying the key symptoms that significantly impact diagnostic conclusions is essential for informed clinical decision-making and personalized treatment planning [26,27].

For the above reasons, within the scope of this study, a new multi-modal data fusion with fuzzy knowledge graph approach is developed to address the integration of medical imaging data and electronic health records (EHRs) for enhancing the accuracy of diagnosis against the uni-modal approaches. It is then applied for supporting ophthalmological disease diagnosis. The contributions and novelties of this paper are shown as follows:

- **Proposing a new multi-modal data integration framework**: A novel framework for multi-modal data fusion has been introduced, grounded in the principles of fuzzy rule-based systems and fuzzy knowledge graphs (FKG). This framework is specifically engineered to effectively manage the challenges posed by the high dimensionality and heterogeneity inherent in multi-modal data, thereby rendering it highly applicable to complex domains such as healthcare.
- **Proposing a method to fuse tabular data and image data in the medical field**: This technique extracts important image features integrated with tabular data features to increase confidence in disease diagnosis.
- **A demonstrative experiment in the case study of ophthalmological disease**: Comparative experiments are conducted on the benchmark medical BRSET dataset, which comprises retinal fundus images and patient metadata, to predict demographic characteristics and diabetic retinopathy disease detection. This shows the applicability of the proposed method.

From an academic perspective, this paper proposes a framework that introduces novel techniques for fusing features from both image and tabular data, two commonly used data types in the healthcare domain. From a practical application standpoint, the paper demonstrates that the proposed model is both suitable and effective in integrating diverse data types to enhance the diagnosis of diabetic retinopathy.

## Related works

### Data modalities in healthcare

**Fig 1:**
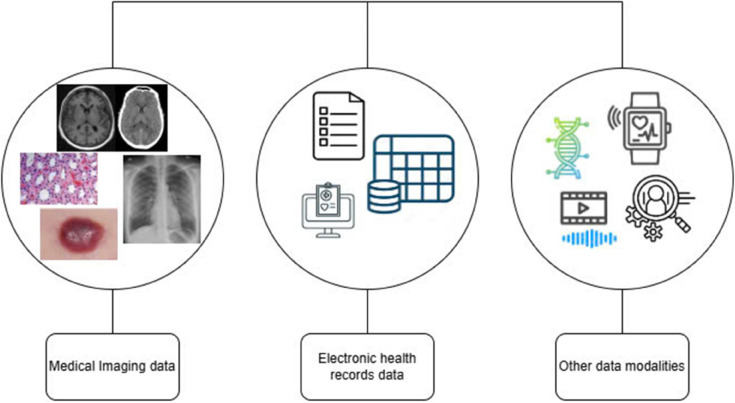
Data modalities in healthcare.

Data modalities in healthcare refer to the different types or forms of data that are collected, generated, and used within the healthcare system to monitor, diagnose, and treat patients. Healthcare encompasses a wide range of data modalities, as illustrated in Fig 1. Through appropriate data processing techniques, such as extracting features from medical images, structuring electronic health records (EHRs), and analyzing data from wearable devices, this raw information is transformed into structured, meaningful insights that can support clinical analysis and decision-making [28].

#### Medical imaging data.

Medical imaging serves a vital function in healthcare by offering essential diagnostic insights and supporting the management of a broad spectrum of medical conditions [14,29–31]. It utilizes sophisticated imaging technologies to produce high-resolution visual representations of internal anatomical structures, thereby enabling doctors and clinicians to identify abnormalities, assess disease progression, and guide therapeutic interventions. Nowadays, medical imaging is progressively being combined with advanced data analytics and digital technologies, contributing to greater diagnostic precision, increased operational efficiency, and expanded access to healthcare services [32].

#### Electronic Health Records (EHRs).

Digital Medical Records function as a centralized archive of patient medical data, facilitating information access by healthcare providers [14,33]. The widespread adoption of EHR systems has led to a substantial growth in both the volume and complexity of patient-related data [34]. Despite their richness and patient-specific nature, these datasets are frequently fragmented and lack a standardized structure. These datasets involve a wide range of variables, including medication histories, laboratory results, imaging findings, physiological measurements, and clinical notes [35,36], thereby introducing analytical challenges due to their heterogeneity and complexity. Machine learning (ML) techniques offer a promising approach to address these challenges by uncovering complex, non-linear relationships within the diverse variables embedded in EHR datasets [37].

#### Other data modalities in healthcare.

Wearable devices have gained significant prominence in the healthcare field, offering promising capabilities for continuous monitoring and assessment of various health and wellness indicators. Typically designed for wear on the body or embedded in clothing and accessories, these devices capture real-time data on sleep behavior, physical activity, vital signs, and additional health indicators [38]. This data modality provides crucial understanding of an individual’s comprehensive health status, supporting customized health surveillance and the strategies for the prevention of disease [39]. Furthermore, wearable technologies enable remote patient monitoring, allowing healthcare professionals to track patients’ health conditions from a remote location and take appropriate action when required.

Similarly, sensor data is pivotal in the advancement of smart healthcare, as it allows for the live monitoring of physiological indicators and daily routines. The continuous collection of sensor data aids in the early identification and prompt intervention of potential health issues [40]. This form of data collection provides healthcare providers with objective and accurate information, enhancing clinical decision-making and enabling the development of personalized treatment regimens. For instance, sensor technologies are instrumental in managing chronic diseases such as diabetes or cardiovascular disorders through continuous monitoring of variables like blood glucose concentrations and heart rate variability. Real-time sensor data is also fundamental to telemedicine, digital health services, and remote monitoring of patients, thereby facilitating care delivery for patients with limited mobility or those in geographically isolated regions, reducing the necessity for frequent individual clinical visits [41,42].

Genomic data is crucial to the advancement of healthcare, providing vital insights into a person’s genetic makeup and its influence on health and disease [43]. Recent progress in genomic sequencing technologies has greatly enhanced the accessibility and affordability of acquiring personal genetic information. Genomic data supports a wide range of applications, including the prediction and diagnosis of hereditary conditions, along with the genetic markers identification linked to disease susceptibility and treatment responsiveness [44]. Moreover, it serves as a cornerstone of personalized medicine by informing therapeutic strategies based on an individual’s specific genetic profile [45].

In smart healthcare, environmental data also holds substantial value, understanding the impact of environmental variables on personal health, including variables such as humidity, ambient temperature, noise levels, pollution levels, air quality, and other environmental-specific conditions. Incorporating environmental data into smart healthcare systems enables a more comprehensive understanding of how external conditions may impact patient health results [46]. For instance, air quality monitoring allows for the identification of regions with elevated pollution levels, which is especially beneficial for persons suffering from respiratory diseases such as asthma.

### Data fusion techniques

Data fusion techniques involve the combination of information from various sources to extract meaningful and actionable insights. These methods enhance the accuracy and reliability of inferences beyond what can be achieved through the analysis of individual data sources alone. Accordingly, data fusion aims to aggregate information originating from heterogeneous and, at times, complementary or competing sources, thereby capturing the collaborative dynamics within complex systems [47]. By transforming raw data into knowledge-driven insights, data fusion facilitates more informed and dependable decision-making processes, rendering it a valuable approach in the context of Structural Health Monitoring (SHM) systems [48].

**Fig 2:**
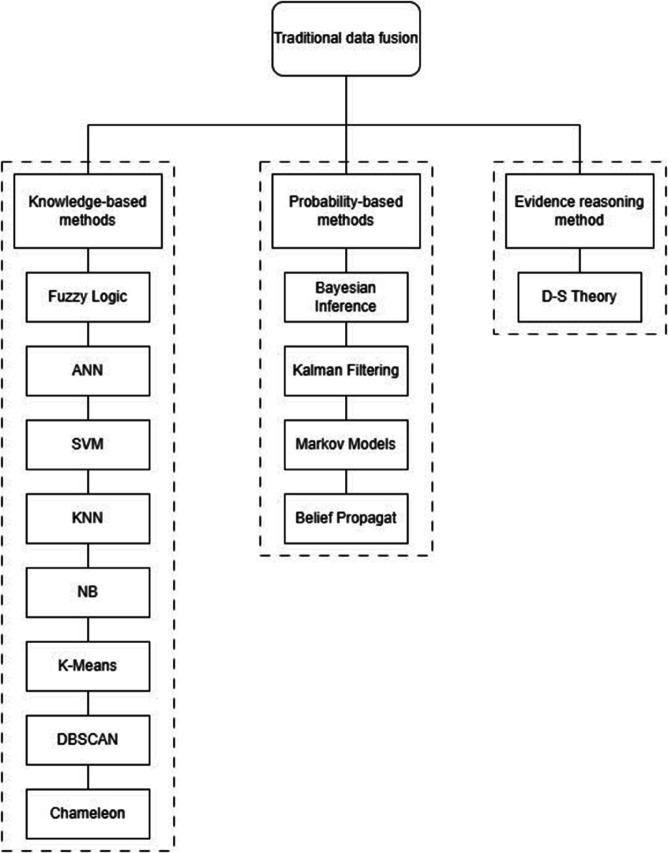
Traditional data fusion.

Over time, a variety of mathematical frameworks have been employed to develop sophisticated data integration algorithms. An extensive introduction and discussion of these methodologies were provided by Meng et al. [49] provided an extensive introduction and discussion of these methodologies. This overview highlights the most widely adopted data fusion techniques, detailing their distinct features, associated challenges, advantages, and limitations within SHM applications. As outlined by Pires et al. [50], traditional data fusion approaches can generally be classified into three main categories: (1) probability-based methods, (2) evidence reasoning approaches, and (3) knowledge-based techniques. These classifications, along with corresponding data fusion strategies, are illustrated in Fig 2 [47].

### Unimodal and Multi-modal data integration models

Diabetic retinopathy (DR) is a retinal disorder resulting from diabetes, predominantly impacting the retina’s structure and function. It represents one of the primary causes of visual impairment and blindness in developed nations [51]. Prior research on ophthalmic diagnostic models has highlighted the significant possible of image recognition-based artificial intelligence in automating tasks traditionally reliant on clinical expertise [52]. However, individual deep learning models often encounter limitations in effectively extracting critical features from complex retinal images. To address this challenge, Khan et al. propose a robust ensemble-based approach for diabetic retinopathy (DR) diagnosis, structured into four primary phases: image pre-processing, selection of pre-trained backbone models, feature enhancement, and optimization. The process begins with image pre-processing, wherein Contrast Limited Adaptive Histogram Equalization (CLAHE) is employed to enhance image contrast. The proposed model was evaluated using the multiclass APTOS 2019 Kaggle dataset, achieving an accuracy of 88.52% [53]. Meanwhile, many AI systems that support the diagnosis of eye diseases in general and diabetic retinopathy in particular have been recognized by prestigious organizations such as the FDA and are widely deployed [54]. Before being applied in practice, these AI systems must undergo an evaluation and verification process for algorithms and data [55]. This verification can be done by a team of experts in the field of ophthalmology or based on previously certified AI products [56].

The fusion of multi-modal medical data has become a revolutionary approach in the field of medicine, facilitating a more holistic insight into patient health conditions and enabling the development of personalized treatment strategies [28]. The process of constructing a multi-modal deep learning framework in healthcare generally adheres to the conventional machine learning pipeline, encompassing stages such as problem formulation, data preprocessing, model training, and performance evaluation [3]. Healthcare researchers are increasingly leveraging the benefits of multi-modality to improve clinical outcomes. For example, Pingali, L. introduced a multi-modal machine learning approach for predicting the progression of knee osteoarthritis by combining clinical data with plain radiographs [57]. Similarly, the study in [29] proposed a personalized, multi-modal, and cost-efficient Oral Health Advisor, capable of automatically classifying sensor-derived data and delivering interactive oral health guidance. Collectively, these studies underscore the advantages of multi-modal machine learning, particularly its ability to incorporate diverse data sources and enhance predictive accuracy by capturing a broader set of features through data fusion. Ophthalmology, as a field that extensively depends on multi-modal information, necessitates comprehensive patient histories alongside detailed visual assessments. As a result, multi-modal machine learning is gaining growing significance in advancing diagnostic capabilities within ophthalmic practice [58]. Specifically for diabetic retinopathy, there have been a number of review studies demonstrating the effectiveness and benefits of using multi-modal image data in diagnosing this disease, such as color photography, OCTA, or OCT, etc. [59,60]. In addition, the study by Restrepo et al. [5] in 2024 also conducted experiments on a multi-modal dataset combining image and tabular data. Therefore, there exists both a significant need and considerable potential for the continued advancement of multi-modal artificial intelligence models to support the diagnosis and triage of ophthalmic diseases.

### Knowledge Graphs (KGs) and Fuzzy Knowledge Graphs (FKGs)

The rapid expansion of AI and big data technologies has underscored the critical need for effective methods of organizing and representing vast volumes of knowledge. Knowledge graphs (KGs) have emerged as a powerful tool for structuring and conveying real-world information through graph-based data models [61]. These characteristics have led to the increasing application of KGs across diverse domains, particularly in healthcare. Nevertheless, traditional KGs face limitations when dealing with datasets that contain ambiguous, incomplete, or uncertain information.

To address these challenges, Fuzzy Knowledge Graphs (FKGs) have recently emerged as a promising extension of KGs, combining fuzzy logic with approximate reasoning to improve inference capabilities in uncertain environments [23]. Introduced in 2020, FKGs integrate fuzzy inference mechanisms to identify implicit relationships and derive novel labels that cannot be inferred through conventional rule-based systems. Although the M-CFIS-FKG model proposed by Lan et al. has addressed several limitations inherent in traditional knowledge graphs, it remains constrained by its reliance on single entity pairs during the inference process. To overcome this limitation, Long et al. [24] introduced an enhanced model known as the pair-form fuzzy knowledge graph (FKG-Pairs) for the diagnosis of preeclampsia symptoms in pregnant women, aiming to facilitate decision-making in clinical settings where input datasets may be partially incomplete [62]. The effectiveness of the FKG-Pairs3 model has been demonstrated through its application in approximate reasoning for disease diagnosis within the domain of traditional medicine [63]. Furthermore, Long et al. [25] developed the FKG-Extreme model to support decision-making in complex or extreme cases, successfully applying it to the diagnosis of chronic kidney disease, demonstrating its utility in handling challenging medical scenarios [23,64].

Although FKG-based models have demonstrated strong capabilities in representing uncertain knowledge, a key limitation lies in their reliance on restricted datasets derived from one-source data. To address this constraint, a study of Tan et al. [65] introduced a novel conceptual framework, termed FKG-S, which integrates data from multiple sources. However, this paper has some limitations, such as only using unimodal datasets, not enhancing the multi-source data integration module to support the integration of diverse input data types, or not showing the integration strategies using many data fusion techniques to effectively combine heterogeneous data modalities.

## The proposed FKG-MM framework

In this section, the proposed FKG-MM framework is introduced for integrating multi-modal data based on the fuzzy knowledge graph model FKG applied to the medical field. Firstly, the general framework is described step by step from data collection to model representation and output. Next, the solution to support the diagnosis of diabetic retinopathy based on tabular data and medical image data is described.

### The FKG-MM framework

**Fig 3:**
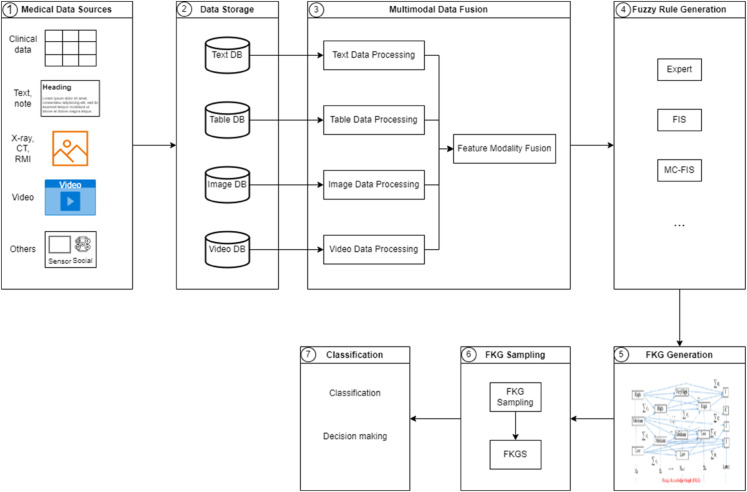
The proposed FKG-MM framework.

The FKG-MM framework is based on the FKG model to integrate multi-modal data, such as image data, tabular data, text data, and EEG signal data, to support highly effective disease diagnosis. The framework consists of several components, with the first part being the component that collects and stores data from various sources and different data models. Next, the data is preprocessed, fusing different types of data as a basis for model building. The final component is the predictive and diagnostic model that gives the results. The components of the FKG-MM framework are shown in Fig 3 and are described in detail as follows:

1. **Data Sources:** In the era of big data, data comes from many sources and exists in many different models. In the field of data, it can be structured or unstructured, but it often has common forms such as data images, data texts, time series data, data tables, data videos, etc. These types of data are collected through the examination process or medical devices. Input data sources play an important role in supporting patient diagnosis and treatment.
2. **Data storage:** In this step, data collection modules are designed to gather data relevant to specific contexts, identify the data types of each dataset, and deliver them to the corresponding storage space, and automatically store data in a distributed storage system and segregate different types of data (text, images, videos, and audio) accordingly.
3. **Multi-modal data fusion:** Once the data is collected, multi-modal AI systems fuse the data types together. Unlike unimodal systems, multi-modal models use architectures that process multiple data formats simultaneously. For example, a model might analyze both the pixels of an image and its associated text annotations to better understand the visual content. This approach takes advantage of complementary strengths: text provides descriptive context, while images provide spatial and visual detail. To fuse these data types, multi-modal systems can use different strategies such as early fusion, late fusion, intermediate fusion, or concatenate data features. This step is the most crucial step of the proposed model, where instead of processing raw data, feature extraction and data fusion are performed. Details of this phase will be presented in the next section.
4. **Fuzzy rule generation:** This function utilizes common fuzzy logic mechanisms, such as FIS or expert knowledge, to transform the knowledge stored in the data repository into a fuzzy rule base (FRB).
5. **FKG generation:** FKG is a type of knowledge graph that adds fuzzy relationships on the edges. The input of this module is the fuzzy rule base. Lan et al. [23] in 2020 published an algorithm to build FKG from FRB. Later, Long et al. [24] improved the FKG representation model into the FKG-Pairs model.
6. **FKGS sampling:** Although FKG is suitable and effective when applied to datasets with missing or uncertain properties. However, due to the complexity of representation and calculation, the FKG model is difficult to apply to huge data sets. Graph sampling is an effective approach for FKG modeling when the input data set is large. Within the model, a purposeful random sampling algorithm is employed on the FKG to generate a sample set of FKGs, referred to as FKGS, also known as FKGS. FKGS has all the basic properties of FKG. Instead of being calculated on FKG, only FKGS needs to be calculated. This significantly reduces the computational cost.
7. **Classification:** In the classification module, the FISA algorithm [23] is utilized to compute on FKGS for the purpose of classification or aiding in decision-making.

As shown above, a multi-modal data integration framework based on the FKG fuzzy knowledge graph model is described to improve the performance and reliability of disease classification diagnosis. However, in the medical field, there are many different types of diseases, each with a different set of symptoms, so that effective diagnosis requires different input from examination data sources. In addition, the FKG and FKGS models have proven their effectiveness when applied to disease diagnosis with a single data source in tabular form. For that reason, the FKG-MM framework was introduced with the aim of integrating image data with tabular data to diagnose Diabetic Retinopathy. The next section will present this integration model in detail.

### A case study of FKG-MM for diabetic retinopathy disease diagnosis

**Fig 4:**
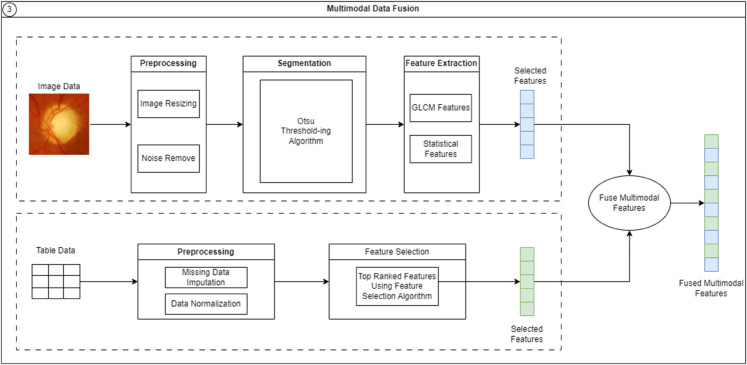
FKG-MM for diagnosis of diabetic retinopathy diseases.

This section describes the application of FKG-MM for the classification of diabetic retinopathy diseases. The process is comprised of three main stages: initially, image feature extraction is conducted; subsequently, feature selection from tabular data is performed; and finally, the extracted image features are integrated with the selected tabular data features. Fig 4 depicts an overview of the proposal strategy. However, in real-world scenarios, the process of data collection and preparation often involves potential conflicts between different types of data. In such cases, it is necessary to perform disagreement checks to assess the degree of inconsistency, which helps determine the feasibility of unifying the data before proceeding with integration.

#### Processing on medical image modality:

1. **Data preprocessing.** As a crucial initial stage, preprocessing is applied to retinal fundus images to reduce noise and variability while enhancing image quality and contrast. Beyond contrast enhancement and noise suppression, this step also facilitates image normalization and correction of non-uniform illumination, helping to minimize artifacts and boost the accuracy of subsequent processing stages. Additionally, diabetic eye disease (DED) features are localized, extracted, and segmented from the fundus images to enable more accurate classification using pre-trained models.
  *Image Resizing.* Initially, a grayscale transformation is performed to convert input RGB images into grayscale images. A grayscale image contains only shades of gray, with no presence of red, green, or blue. Using grayscale images simplifies image processing tasks and reduces complexity.
  *Noise Removal.* Digital images may be affected by noise during acquisition, transmission, or subsequent processing stages. To mitigate or eliminate such noise, a variety of filtering techniques—such as Gaussian blurring, median blurring, mean blurring, and bilateral filtering—are commonly employed. To blur or remove noise, various filtering techniques such as mean blur, median blur, Gaussian blur, and bilateral filtering are commonly used. This process adjusts digital images to improve subsequent analysis and aid in the identification of important features. Image enhancement is performed through histogram equalization, which improves the contrast of the image.
2. **Segmentation.** Segmentation primarily focuses on identifying similar regions within an image and dividing objects into distinct areas based on a threshold value. One of the most effective image segmentation techniques is Otsu’s thresholding, a histogram-based global thresholding method. This technique assumes that the image consists of two pixel categories (foreground and background) based on a bi-modal histogram. It determines the optimal threshold that that enhances the differences between classes or, equivalently, reduces the variance within each class. Otsu’s thresholding is a nonlinear method for converting a grayscale image into a binary one. The largest segmented object is selected for further analysis, while smaller objects are removed to prevent inaccurate results. The image preprocessing and segmentation operations are described in detail in Algorithm 1. **Image feature extraction.** Feature extraction is a fundamental step in the analysis and identification of relationships among objects. Because image prediction, categorization, and recommendation algorithms cannot process images in their raw form, feature extraction is necessary to convert images into an interpretable format. This increases classifier complexity and computational load when processing irrelevant features, reducing classification accuracy. To achieve precise image classification, it is necessary to extract sufficient relevant features. Segmenting images and extracting multiple features from different regions is the most effective approach. In this study, texture classification was performed using GLCM features, while various statistical features were employed to analyze color information for disease classification. Algorithm 2 details the feature extraction method.
  *GLCM Features [66].* Initially, each image is processed through the Gray Level Co-occurrence Matrix (GLCM) method. The extracted GLCM features, along with their corresponding descriptions, are presented below:
  - Contrast reflects the spatial frequency of an image and a moment of the GLCM, describing the difference between adjacent pixel values. It measures local variations, where low contrast results in GLCM values concentrated near the main diagonal, indicating low spatial frequencies.
  - Homogeneity, or inverse difference moment, measures image uniformity and is higher when gray-level differences between pixel pairs are small. It is sensitive to elements near the GLCM diagonal and peaks when all pixel values are identical. Homogeneity inversely correlates with contrast: as contrast increases, homogeneity decreases, with energy remaining constant.
  - Energy is the square root of the angular second moment and increases when the image shows greater uniformity or structure.
  - Entropy measures the degree of randomness and uniformity among pixels within an image.
  - Correlation quantifies the degree of association between a pixel and its neighboring pixels across the entire image.
  - Angular Second Moment (ASM), which measures textural uniformity through pixel pair repetitions and detects texture irregularities. Its maximum value is 1, with higher values indicating a constant periodic gray-level pattern.

**Algorithm 1 Image preprocessing and segmentation.**

1: **function** UNSHARP_MASK(image)      ▷ Sharpen image using unsharp masking technique

2:   blurred ← GaussianBlur(image)

3:   sharpened ← image × (1 + α) – blurred × α

4:   **return** sharpened

5: **end function**

6: **function** APPLY_CLAHE(image)      ▷ Increase local contrast

7:   lab_image ← convert image to LAB color space

8:   *l*,*a*,*b* ← split lab_image

9:   clahe ← CLAHE object (clipLimit = 2.0, tileGridSize = (8,8))

10:   l_clahe ← apply CLAHE to *l*

11:   merged ← merge l_clahe, *a*, *b* and convert to BGR

12:   **return** merged

13: **end function**

14: **function** DENOISE_IMAGE(image)      ▷ Denoising using fast Non-local Means Denoising

15:   denoised ← apply fastNlMeansDenoisingColored

16:   **return** denoised

17: **end function**

18: **function** NORMALIZE_NUMERIC_FEATURES(dataframe)      ▷ Normalize numeric columns to the range [0, 1] or by Z-score

19:   **for** column in dataframe **do**

20:    **if** column is numeric **then**

21:     Apply Min-Max Scaling or StandardScaler

22:    **end if**

23:   **end for**

24:   **return** dataframe

25: **end function**

26: **function** PREPROCESS_IMAGE(image)      ▷ Image preprocessing pipeline

27:   sharpened ← UNSHARP_MASK(image)

28:   denoised ← DENOISE_IMAGE(sharpened)

29:   clahe_image ← APPLY_CLAHE(denoised)

30:   gray ← convert clahe_image to grayscale

31:   lesion_mask ← apply Otsu thresholding on gray

32:   **return** (clahe_image, lesion_mask)

33: **end function**

**Algorithm 2 Image features extraction.**

1: **function** EXTRACT_GLCM_FEATURES(image_folder)      ▷ Extract texture features (GLCM) from retinal images

2:   Initialize empty list glcm_features

3:   image_paths ← list all image files in image_folder

4:   **for** each path in image_paths **do**

5:    image ← read and resize image

6:    (enhanced_image, lesion_mask) ← PREPROCESS_IMAGE(image)

7:    variance_feature ← calculate_variance(data)

8:    std_dev_feature ← calculate_standard_deviation(data)

9:    rms_feature ← calculate_rms(data)

10:    mean_feature ← calculate_mean(data)

11:    Append (variance_feature, std_dev_feature, rms_feature, mean_feature) to features_arr

12:    gray_image ← convert enhanced_image to grayscale

13:    masked_image ← apply lesion_mask on gray_image

14:    normalized_image ← rescale pixel values into 16 gray levels

15:    glcm ← compute GLCM (distances = [1], angles = [$0^{\circ }$, $45^{\circ }$, $90^{\circ }$, $135^{\circ }$])

16:    features ← extract contrast, homogeneity, correlation, etc. from glcm features

17:    Append features to features_arr

18:   **end for**      ▷ Normalize continuous features from images

19:   columns_to_normalize ← [“Variance Feature”, “Standard Deviation Feature”, “RMS Feature”, “Mean Feature”]

20:   **for** col in columns_to_normalize **do**

21:    **if** col ∈ features_arr.columns **then**

22:     features_arr[col] ← MIN_MAX_SCALING(features_arr[col])

23:    **end if**

24:   **end for**

25:   **return** features_arr

26: **end function**

Each of the listed GLCM features is computed by the Eqs 1 to 6:

$$\text{Contrast}=\sum_{i=0}^{N-1}\sum_{j=0}^{N-1}(i-j{)}^{2}P(i,j)$$ (1)

$$\text{Homogeneity}=\sum_{i=0}^{N-1}\sum_{j=0}^{N-1}\frac{P(i,j)}{1+(i-j{)}^{2}}$$ (2)

$$\text{Energy}=\sqrt{\sum_{i=0}^{N-1}\sum_{j=0}^{N-1}P(i,j{)}^{2}}$$ (3)

$$\text{Entropy}=\sum_{i=0}^{N-1}\sum_{j=0}^{N-1}\left(-\ln(P(i,j))P(i,j)\right)$$ (4)

$$\text{Correlation}=\sum_{i=0}^{N-1}\sum_{j=0}^{N-1}P(i,j)\frac{\left(i-\mu_{i})(j-\mu_{j}\right)}{\sqrt{\sigma_{i}\sigma_{j}}}$$ (5)

$$\text{ASM}=\sum_{i=0}^{N-1}\sum_{j=0}^{N-1}P(i,j{)}^{2}$$ (6)

where *P*(*i*, *j*) represents the symmetrically normalized GLCM, *N* denotes the total number of gray levels in the image, $\mu_{i}$ and $\mu_{j}$ are the means of the $i^{\text{th}}$ row and $j^{\text{th}}$ column as in Eqs 7, 8 and $\sigma_{i}$, $\sigma_{j}$ correspond to the variances of the $i^{\text{th}}$ row and $j^{\text{th}}$ column as in Eqs 9, 10.

$$\mu_{i}=\sum_{i=0}^{N-1}i\sum_{j=0}^{N-1}P(i,j)$$ (7)

$$\mu_{j}=\sum_{j=0}^{N-1}j\sum_{i=0}^{N-1}P(i,j)$$ (8)

$$\sigma_{i}=\sqrt{\sum_{i=0}^{N-1}(i-\mu_{i}{)}^{2}\sum_{j=0}^{N-1}P(i,j)}$$ (9)

$$\sigma_{j}=\sqrt{\sum_{j=0}^{N-1}(j-\mu_{j}{)}^{2}\sum_{i=0}^{N-1}P(i,j)}$$ (10)

Next, statistical features are extracted [67]. In this study, mean, root mean square, standard deviation, and variance are extracted as statistical features. The descriptions and formulas of the extracted features are provided below

- Mean: The mean represents the average value of the color intensities across all pixels within the image.
- Variance. The variance of an image measures how spread out the values are around the mean.
- Standard Deviation. The standard deviation is calculated as the square root of the variance of the distribution.
- Root Mean Square. The RMS is calculated as the square root of the mean of all squared intensity values.

Each of the listed statistical features is computed by the Eqs 11 to 14:

$$\text{Mean}=\sum_{i=0}^{N-1}\sum_{j=0}^{N-1}i\cdot P(i,j)$$ (11)

$$\text{Variance}=\sum_{i=0}^{N-1}\sum_{j=0}^{N-1}{\left(i-\mu \right)}^{2}P(i,j)$$ (12)

$$\text{SD}=\sqrt{\sum_{i=0}^{N-1}\sum_{j=0}^{N-1}{\left(i-\mu \right)}^{2}P(i,j)}$$ (13)

$$\text{RMS}=\sqrt{\sum_{i=0}^{N-1}\sum_{j=0}^{N-1}{\left(i-c\right)}^{2}P(i,j)}$$ (14)

#### Processing on medical tabular record modality:

1. **Tabular Data preprocessing.** To ensure data quality, basic preprocessing operations for tabular data, such as noise and outlier removal, handling missing values, and eliminating duplicate data, are performed. After preprocessing, relevant attributes in the table that significantly impact the output label are selected. The specific steps to perform tabular data preprocessing are described in Algorithm 3.
2. **Tabular feature selection.** First, Pearson correlation was used to remove highly correlated features and those with weak linear relationships by setting a correlation threshold, thus reducing dimensionality and mitigating the curse of dimensionality. Next, mutual information was used on the remaining features to identify those with strong dependencies on the target variable. Mutual information, based on entropy, quantifies the information shared between variables without assuming a specific data distribution. This two-step feature selection process helped retain only the most relevant features for further analysis.

**Algorithm 3 Tablular data preprocessing.**

1: **function** LOAD_SELECTED_COLUMNS(file_path, columns)

2:   df ← ReadCSV(file_path)

3:   df ← df[columns]

4:   **return** df

5: **end function**

6: **function** MAP_CATEGORICAL_VALUES(df, mappings)

7:   **for all** (col, map_dict) ∈ mappings **do**

8:    df[col] ← Map(df[col], map_dict)

9:   **end for**

10:   **return** df

11: **end function**

12: **function** PREPROCESS_NUMERIC_COLUMNS(df, numeric_cols)

13:   **for all** col ∈ numeric_cols **do**

14:    df[col] ← ConvertToNumeric(df[col])

15:    df[col] ← FillNAWithMean(df[col])

16:   **end for**

17:   **return** df

18: **end function**

19: **function** REMAP_TARGET_LABELSdf, target_col, label_map

20:   df[target_col] ← Map(df[target_col], label_map)

21:   **return** df

22: **end function**

23: **function** BALANCE_DATAdf, target_col, method

24:   features ← Drop(df, target_col)

25:   labels ← df[target_col]

26:   balancer ← method(random_state=42)

27:   $(X_{res},y_{res})\leftarrow$ FitResample(balancer, features, labels)

28:   **return** Concat(*X*_*res*_, *y*_*res*_)

29: **end function**

30: **function** PROCESS_TABULAR_DATA(file_path, columns, cat_mappings, num_cols, target_col, label_map, balance_method, corr_threshold)

31:   df ← LOAD_SELECTED_COLUMNS(file_path, columns)

32:   df ← MAP_CATEGORICAL_VALUES(df, cat_mappings)

33:   df ← PREPROCESS_NUMERIC_COLUMNS(df, num_cols)

34:   df ← REMAP_TARGET_LABELS(df, target_col, label_map)

35:   df_balanced ← BALANCE_DATA(df, target_col, balance_method)

36:   **return** df_balanced

37: **end function**

#### Multi-modal feature fusion from medical features:

Current multi-modal fusion methods, such as early or intermediate fusion and various feature combination techniques (e.g., concatenation or learning-based), often depend on deep learning methods, which inflates feature dimensions, especially with image data, making meaningful feature selection difficult. Moreover, feature imbalance between data types (e.g., tabular vs. image) complicates integration. Our approach uses simple mathematical operations and carefully selected features from preprocessed data, reducing complexity and enhancing interpretability compared to the deep learning-based methods, which often lack generalizability in diverse medical contexts.

Below are five of the methods that can be used to combine image features and tabular data features.

1. **Feature Selection Fusion:** This method evaluates the importance of each individual feature and only retains the most important features from both sources. It’s a simple but effective approach to eliminate redundant data and reduce the dimension of the feature vector.
  **Algorithm:**
  (a) **Step 1.** Calculate importance scores for each feature in *F*_*img*_ and *F*_*tab*_ (e.g., using mutual information, variance, or feature importance from models like Random Forest).
  (b) **Step 2.** Sort features by importance scores in descending order.
  (c) **Step 3.** Select the *k*_*img*_ and *k*_*tab*_ most important features from each source.
  (d) **Step 4.** Combine the selected features into the *F*_*fused*_ vector.
  The feature selection method is described specifically in Algorithm 4.
**Algorithm 4 FeatureSelectionFusion.**
1: **function** FEATURESELECTIONFUSION(Fimg, Ftab, k_img, k_tab)
2:   img_scores ← COMPUTEFEATUREIMPORTANCE(Fimg)
3:   tab_scores ← COMPUTEFEATUREIMPORTANCE(Ftab)
4:   sorted_img_indices ← SORTINDICESDESCENDING(img_scores)
5:   sorted_tab_indices ← SORTINDICESDESCENDING(tab_scores)
6:   selected_img_indices ← sorted_img_indices[1:k_img]
7:   selected_tab_indices ← sorted_tab_indices[1:k_tab]
8:   $F_{fused}\leftarrow$ [ ]
9:   **for all** i∈ selected_img_indices **do**
10:    Append(*F*_*fused*_, *F*_*img*_*[i]*)
11:   **end for**
12:   **for all** j∈ selected_tab_indices **do**
13:    Append(*F*_*fused*_, *F*_*tab*_*[j]*)
14:   **end for**
15:   **return** *F*_*fused*_
16: **end function**
  **Illustrative example 1.** Suppose there is an input set consisting of
  $$F_{\text{img}}=[f_{\text{img}1},f_{\text{img}2},f_{\text{img}3},f_{\text{img}4},f_{\text{img}5}]\;F_{\text{tab}}=[f_{\text{tab}1},f_{\text{tab}2},f_{\text{tab}3},f_{\text{tab}4}]\;k_{\text{img}}=3\;k_{\text{tab}}=2$$
  By applying the algorithm step by step, the result is obtained:
  **Step 1:** Suppose the importance of the attributes can be calculated as follows:
  img_scores=[0.7,0.3,0.8,0.5,0.4]tab_scores=[0.6,0.9,0.3,0.7]
  **Step 2:** Sort the indices in descending order based on scores:
  sorted_img_indices=[2,0,3,4,1]sorted_tab_indices=[1,3,0,2]
  **Step 3:** Select the top-*k* indices:
  selected_img_indices=[2,0,3]selected_tab_indices=[1,3]
  **Step 4:** Initialize an empty fused feature list:
  $$F_{\text{fused}}=[\;]$$
  Add selected image features to the fused list:
  $$F_{\text{fused}}=[f_{\text{img}3}]\;F_{\text{fused}}=[f_{\text{img}3},f_{\text{img}1}]\;F_{\text{fused}}=[f_{\text{img}3},f_{\text{img}1},f_{\text{img}4}]$$
  Add selected tabular features to the fused list:
  $$F_{\text{fused}}=[f_{\text{img}3},f_{\text{img}1},f_{\text{img}4},f_{\text{tab}2}]\;F_{\text{fused}}=[f_{\text{img}3},f_{\text{img}1},f_{\text{img}4},f_{\text{tab}2},f_{\text{tab}4}]$$
**Output.**
  After being calculated, the output result shows the selection of 3 image features and 2 tabular data features with the highest level of influence.
  $$F_{\text{fused}}=[f_{\text{img}3},f_{\text{img}1},f_{\text{img}4},f_{\text{tab}2},f_{\text{tab}4}]$$
2. **Tensor Product Fusion:** This method uses the Tensor Product to model interactions between all feature pairs from the two data sources. This allows capturing complex non-linear relationships that simpler methods cannot detect. To handle the large dimensionality issue, the method uses a low-rank approximation.
  **Algorithm:**
  (a) **Step 1.** Normalize the features.
  (b) **Step 2.** Compute the Tensor Product between the two feature sets.
  (c) **Step 3.** Use SVD decomposition to reduce the dimensionality of the resulting tensor.
  (d) **Step 4.** Create the final feature representation from the projection matrices.
  The Tensor Product method is described specifically in Algorithm 5.
**Algorithm 5 Tensor product fusion.**
1: **function** TENSOR_PRODUCT_FUSION(Fimg, Ftab, rank)
2:   normalizedFimg ← NORMALIZE(Fimg)
3:   normalizedFtab ← NORMALIZE(Ftab)
4:   fullTensorProduct ← TENSORPRODUCT(normalizedFimg, normalizedFtab)
5:   U,S,V← TRUNCATEDSVD(fullTensorProduct, rank)
6:   sqrtS ← COMPUTESQRTDIAGONAL(S)
7:   Pimg ←U× sqrtS
8:   Ptab ←V× sqrtS
9:   imgProjection ← normalizedFimg × Pimg
10:   tabProjection ← normalizedFtab × Ptab
11:   fusedFeatures ← Concatenate(imgProjection, tabProjection)
12:   **return** fusedFeatures
13: **end function**
  **Illustrative example:** It is supposed that there is:
  - An image feature matrix $F_{\text{img}}\in {\mathbb{R}}^{2\times 3}$ (2 samples, each with 3 features)
  - A text feature matrix $F_{\text{tab}}\in {\mathbb{R}}^{2\times 2}$ (2 samples, each with 2 features)
$$F_{\text{img}}=[\begin{array}{ccc} 4 & 2 & 8\\ 6 & 1 & 3 \end{array}],\;F_{\text{tab}}=[\begin{array}{cc} 5 & 3\\ 9 & 2 \end{array}]$$
  Applying the Tensor algorithm (see example 2 in the Appendix Illustrative example 2 for details), the following result is obtained.
**Result.**
$$\text{fusedFeatures}=[\begin{array}{cccc} 1.157 & -0.276 & 1.386 & 0.079\\ 1.053 & 0.356 & 1.384 & -0.052 \end{array}]$$
  The final fused feature matrix has dimensions [2×4], where:
  - The first two columns contain the projected image features.
  - The last two columns contain the projected text features.
3. **Hadamard Product Fusion:** This method projects features from both sources into a common space of the same dimension, then applies the Hadamard product (element-wise multiplication) to capture direct interactions between corresponding components. This ensures that the integrated feature exploits correlations between aligned features.
  **Algorithm:**
  (a) **Step 1.** Project features from both sources into a common space of the same dimension.
  (b) **Step 2.** Normalize the projected features.
  (c) **Step 3.** Compute the Hadamard product (element-wise multiplication).
  (d) **Step 4.** Apply a non-linear transformation and combine with the original projected features.
  The Tensor Product method is described specifically in Algorithm 6.
**Algorithm 6 Hadamard product fusion.**
1: **function** HADAMARD_PRODUCT_FUSION(Fimg, Ftab, commonDim)
2:   Wimg ← INITIALIZEMATRIX(*p*, commonDim)
3:   Wtab ← INITIALIZEMATRIX(*q*, commonDim)
4:   imgProjected ← Fimg × Wimg
5:   tabProjected ← Ftab × Wtab
6:   imgNormalized ← L2NORMALIZE(imgProjected)
7:   tabNormalized ← L2NORMALIZE(tabProjected)
8:   hadamardProduct ← ELEMENTWISEMULTIPLY(imgNormalized, tabNormalized)
9:   activatedFeatures ← RELU(hadamardProduct)
10:   fusedFeatures ← CONCATENATE(activatedFeatures, imgNormalized, tabNormalized)
11:   fusedFeatures ← LINEARPROJECTION(fusedFeatures, commonDim)
12:   **return** fusedFeatures
13: **end function**
  **Illustrative example:** It is supposed that there is:
  - Image feature matrix $F_{\text{img}}\in {\mathbb{R}}^{2\times 3}$ (2 samples, each with 3 features)
  - Tabular feature matrix $F_{\text{tab}}\in {\mathbb{R}}^{2\times 2}$ (2 samples, each with 2 features)
  - Common dimension: commonDim=2
  Matrices are:
  $$F_{\text{img}}=[\begin{array}{ccc} 1 & 2 & 3\\ 4 & 5 & 6 \end{array}]\;,\;F_{\text{tab}}=[\begin{array}{cc} 7 & 8\\ 9 & 10 \end{array}]$$
  Applying the Hadamard algorithm (see example 3 in the Appendix [Illustrative example 3]Illustrative example 3 for details), the following result is obtained.
  **Result.** The final output of the Hadamard Product Fusion algorithm is:
  $$\text{fusedFeatures}=[\begin{array}{cc} 1.915 & 1.485\\ 1.914 & 1.487 \end{array}]$$
  This fused representation combines information from both image and tabular features, capturing the interactions between corresponding dimensions through the Hadamard product.
4. **Filter-based multi-modal Feature Selection:** Filter-based feature selection evaluates each feature independently using statistical measures, without involving the learning algorithm. multi-modal fusion selects the most relevant features from each modality (image and tabular) while considering both intra-modal redundancy and inter-modal correlation.
  **Algorithm:**
  (a) **Step 1.** Calculate feature importance scores for each feature in Fimg and Ftab using multiple criteria (mutual information and random forest importance).
  (b) **Step 2.** Rank features by importance scores within each modality.
  (c) **Step 3.** Select a larger initial set of candidate features from each modality.
  (d) **Step 4.** Remove highly correlated features within each modality to reduce redundancy.
  (e) **Step 5.** Analyze cross-modal correlation to ensure complementary information.
  (f) **Step 6.** Combine the selected features from both modalities to form *F*_*fused*_.
  The Filter-based multi-modal Feature Selection method is described specifically in Algorithm 7.
**Algorithm 7 Filter multi-modal selection.**
1: **function** FILTER_MULTI-MODAL_SELECTION(Fimg, Ftab, target, k_img, k_tab)
2:   imgScores ← COMPUTEFEATUREIMPORTANCE(Fimg, target)
3:   tabScores ← COMPUTEFEATUREIMPORTANCE(Ftab, target)
4:   sortedImgIndices ← SORTINDICESDESCENDING(imgScores)
5:   sortedTabIndices ← SORTINDICESDESCENDING(tabScores)
6:   candidateImgIndices ← sortedImgIndices[1:2×k_img]
7:   candidateTabIndices ← sortedTabIndices[1:2×k_tab]
8:   finalImgIndices ← REMOVECORRELATEDFEATURES(Fimg, candidateImgIndices, corrThreshold)
9:   finalTabIndices ← REMOVECORRELATEDFEATURES(Ftab, candidateTabIndices, corrThreshold)
10:   finalImgIndices ← finalImgIndices[1:k_img]
11:   finalTabIndices ← finalTabIndices[1:k_tab]
12:   fusedFeatures ← CONCATENATE(Fimg[:, finalImgIndices], Ftab[:, finalTabIndices])
13:   **return** fusedFeatures, {finalImgIndices, finalTabIndices}
14: **end function**
**Illustrative example:**
  Given:
  $$F_{\text{img}}=[\begin{array}{ccccc} 5 & 2 & 8 & 6 & 9\\ 7 & 1 & 4 & 3 & 8\\ 6 & 3 & 9 & 5 & 7 \end{array}]\;\text{(5 image features)}$$
  $$F_{\text{tab}}=[\begin{array}{cccc} 3 & 6 & 2 & 7\\ 8 & 5 & 1 & 9\\ 4 & 7 & 3 & 8 \end{array}]\;\text{(4 tabular features)}$$
  $$\text{target}=[\begin{array}{ccc} 0 & 1 & 0 \end{array}]\;\text{(Target variable)}$$
  Other parameters:
  $$k_{\text{img}}=2\;\text{(Number of image features to select)}$$
  $$k_{\text{tab}}=2\;\text{(Number of tabular features to select)}$$
  Applying the Filter algorithm (see example 4 in the Appendix [Illustrative example 4]Illustrative example 4 for details), the following result is obtained.
  **Final Result** The final selected feature set is:
  $$F_{fused}=[img_{3},img_{1},tab_{2},tab_{4}]$$
  The algorithm has selected:
  - 2 image features: *img*_3_ and *img*_1_
  - 2 tabular features: *tab*_2_ and *tab*_4_
  This is the optimal feature set based on importance criteria, with low intra-modal correlation and complementary information across modalities.
5. **Wrapper-based multi-modal Feature Selection:** Wrapper methods assess feature subsets by iteratively training and evaluating a specific model, selecting the subset of features that maximizes the model’s performance. For multi-modal fusion, it performs Sequential Forward Selection (SFS) to incrementally build an optimal feature set from both modalities, directly optimizing for the fusion task.
  **Algorithm:**
  (a) **Step 1.** Define evaluation model (e.g., Random Forest) and performance metric based on task.
  (b) **Step 2.** Initialize empty feature sets for both modalities.
  (c) **Step 3.** Pre-filter features using simpler filter method to reduce search space.
  (d) **Step 4.** Ensure minimum representation from each modality.
  (e) **Step 5.** Perform Sequential Forward Selection, evaluating all potential feature additions.
  (f) **Step 6.** Continue until maximum features are selected or no improvement is seen.
  (g) **Step 7.** Combine the selected features to form *F*_*fused*_.
  The Wrapper-based multi-modal Feature Selection method is described specifically in Algorithm 8.
**Algorithm 8 Wrapper multi-modal selection.**
1: **function** WRAPPERMULTI-MODALSELECTION(Fimg, Ftab, target, max_img, max_tab)
2:   selected_img_indices ← []      ▷ Initialize empty image feature set
3:   selected_tab_indices ← []      ▷ Initialize empty tabular feature set
4:   best_score ← −∞
5:   **for** *i* = 1 to min_img_features **do**
6:    selected_img_indices ← ADDBESTFEATURE(Fimg, selected_img_indices, target)
7:   **end for**
8:   **for** *i* = 1 to min_tab_features **do**
9:    selected_tab_indices ← ADDBESTFEATURE(Ftab, selected_tab_indices, target)
10:   **end for**
11:   **while** len(selected_img_indices) < max_img **or** len(selected_tab_indices) < max_tab **do**
12:    best_new_score ← −∞
13:    best_new_feature ← NULL
14:    best_modality ← NULL
15:    **for** each feature *i* in Fimg not in selected_img_indices **do**
16:     temp_score ← EVALUATEFEATURESET(Concatenate(Fimg[:, selected_img_indices + [i]], Ftab[:, selected_tab_indices]), target)
17:     **if** temp_score > best_new_score **then**
18:      best_new_score ← temp_score
19:      best_new_feature ← *i*
20:      best_modality ← ’img’
21:     **end if**
22:    **end for**
23:    **for** each feature *j* in Ftab not in selected_tab_indices **do**
24:     temp_score ← EVALUATEFEATURESET(Concatenate(Fimg[:, selected_img_indices], Ftab[:, selected_tab_indices + [j]]), target)
25:     **if** temp_score > best_new_score **then**
26:      best_new_score ← temp_score
27:      best_new_feature ← *j*
28:      best_modality ← ’tab’
29:     **end if**
30:    **end for**
31:    **if** best_new_score > best_score **then**
32:     **if** best_modality = ’img’ **then**
33:      selected_img_indices.append(best_new_feature)
34:     **else**
35:      selected_tab_indices.append(best_new_feature)
36:     **end if**
37:     best_score ← best_new_score
38:    **else**
39:     **Break**
40:    **end if**
41:   **end while**
42:   Ffused ← CONCATENATE(Fimg[:, selected_img_indices], Ftab[:, selected_tab_indices])
43:   **return** Ffused, {selected_img_indices, selected_tab_indices, best_score}
44: **end function**
**Illustrative example:**
  Let me provide a step-by-step example illustrating the **Wrapper-based multi-modal Feature Selection** algorithm.
  The following data is going to be used:
  - *F*_*img*_: Image feature matrix with features [img_1, img_2, img_3, img_4, img_5]
  - *F*_*tab*_: Tabular feature matrix with features [tab_1, tab_2, tab_3, tab_4]
  - target: Target variable
  - max_img = 3 (Maximum image features to select)
  - max_tab = 2 (Maximum tabular features to select)
  - min_img_features = 1 (Minimum image features required)
  - min_tab_features = 1 (Minimum tabular features required)
  Applying the Wrapper algorithm (see example 5 in the Appendix [Illustrative example 5]Illustrative example 5 for details), the following result is obtained.
  **Result.** Final selected features:
  - Image features: img_3, img_1, img_5
  - Tabular features: tab_2, tab_4
  *F*_*fused*_ is formed by concatenating these selected features.
  The final feature set is [img_3, img_1, img_5, tab_2, tab_4] with a performance score of 0.85.
  This example demonstrates how the wrapper-based approach systematically evaluates combinations of features from both modalities to find the optimal subset that maximizes the model’s performance on the specific task.

### FKGS generation from FKG and FRB

Rule-based systems function by processing and interpreting information using pre-established rules or logical statements. Inferences can be drawn, and knowledge can be extracted from the provided data through the application of these rules. Fuzzy logic, a mathematical framework designed to manage uncertainty, is utilized by rule-based systems to model and reason with imprecise or uncertain data. Membership degrees are assigned to both antecedents and consequents by fuzzy rules, allowing for more versatile decision-making and acknowledging the intrinsic uncertainty in the fusion of medical data. Fuzzy rules facilitate more flexible and adaptive decision-making by attributing degrees of membership to both antecedents and consequents, thereby effectively representing the inherent uncertainty involved in the integration of medical data.

**Fig 5:**
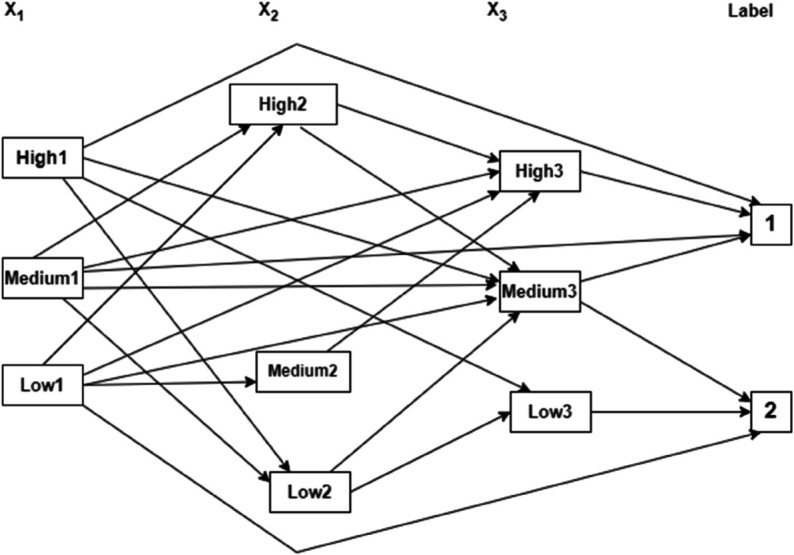
FKG of 6 fuzzy rules [65].

This section describes the process of constructing FKG from FKG and FKGS from FKG through specific examples. As defined by Lan et al. [23], fuzzy knowledge graph FKG is a tuple (V,L,A,B,R), where V is a set of input attributes, each attribute takes on a linguistic variable value; L is a set of output labels; Matrices A and B represent the relationships between the input attribute vertices and the output labels, while R denotes a set of fuzzy rule bases (FRB). For example, an FRB with 6 rules, as shown in Table 1, is given. Applying the algorithm to build a fuzzy knowledge graph by calculating the adjacency matrices A and B, A fuzzy knowledge graph can be built, as shown in Fig 5.

**Table 1:** An illustration of 6 fuzzy rules [23].

Rule	$X_{1}$	$X_{2}$	$X_{3}$	Label
*R*_1_	Medium1	High2	High3	1
*R*_2_	High1	Low2	Low3	2
*R*_3_	Low1	Medium2	High3	1
*R*_4_	Low1	High2	Medium3	1
*R*_5_	High1	Low2	Medium3	2
*R*_6_	Medium1	Low2	Low3	2

FKG is still limited when applied to problems with large data due to the computation time. Tan et al. (2025) [65] proposed a sampling algorithm designed to identify the structure of the fuzzy knowledge graph (FKGS). This is an abstract version of FKG to reduce the computation time but still ensure reliability. FKGS has all the components as in FKG but on a smaller scale. The following example distinguishs and clarifies the process of finding FKGS from FKG. Assume that a collection of 14 fuzzy rules is presented in Table 2, each consisting of 4 attributes and 2 output labels [65]. Sampling algorithm [65] when sample rate 30% and error threshold 0.3 are applied on fuzzy knowledge graph with fuzzy rule base as in Table 2, The obtained FKGS consists of 5 rules. as in Table 3.

**Table 2:** FRB consists of 14 fuzzy rules [65].

Rule	Outlook	Temprateture	Humidity	Windy	Play
*R*_1_	Sunny	Hot	High	FALSE	No
*R*_2_	Sunny	Hot	High	TRUE	No
*R*_3_	Overcast	Hot	High	FALSE	Yes
*R*_4_	Rainy	Mild	High	FALSE	Yes
*R*_5_	Rainy	Cool	Normal	FALSE	Yes
*R*_6_	Rainy	Cool	Normal	TRUE	No
*R*_7_	Overcast	Cool	Normal	TRUE	Yes
*R*_8_	Sunny	Mild	High	FALSE	No
*R*_9_	Sunny	Cool	Normal	FALSE	Yes
*R*_10_	Rainy	Mild	Normal	FALSE	Yes
*R*_11_	Sunny	Mild	Normal	TRUE	Yes
*R*_12_	Overcast	Mild	High	TRUE	Yes
*R*_13_	Overcast	Hot	Normal	TRUE	Yes
*R*_14_	Rainy	Mild	High	TRUE	No

**Table 3:** FKGS consists of 5 fuzzy rules [65].

Rule	Outlook	Temprateture	Humidity	Windy	Play
*R*_3_	Overcast	Hot	High	FALSE	Yes
*R*_6_	Rainy	Cool	Normal	TRUE	No
*R*_8_	Sunny	Mild	High	FALSE	No
*R*_9_	Sunny	Cool	Normal	FALSE	Yes
*R*_11_	Sunny	Mild	Normal	TRUE	Yes

### Advantages and disadvantages of the FKG-MM model

The main characteristic of the proposed framework (FKG-MM) is the ability to integrate data from many different sources and models. Specifically, the FKG model of Lan et al. [23], the FKG-Pairs model of Long et al. [24], FKGS model of Tan et al. [65] calculate on unimodal tabular data sets, while the FKG-MM framework can integrate additional image data components with tabular data to improve the reliability of diagnosis.

The FKG-Integration model has several advantages as follows: (1) The FKG-MM framework shows its suitability for integrating multi-component data in the medical field; (2) The multi-modal data integration module is specifically designed to integrate tabular medical data with image data, which are two of the most common data types in medical diagnosis; (3) The FKG sampling algorithm shows their suitability for the large, and multi-component characteristics of medical data in assisting decision making in disease diagnosis.

## Experimental results

In this section, the experimental results are shown to confirm the effectiveness of the suggested approach. The experiments were carried out to assess how effectively the model performed when combining multi-modal data, which included both images and symptom data, in contrast to using each type of data individually.

### Experimental environments

The experiments are conducted on a system with configurations of HP Victus 16-e0175AX equipped with an AMD Ryzen processor (Family 25 Model 80, 3.3 GHz) and 8GB of RAM for setting up Python 3.11.5. Currently, vast quantities of multi-modal medical data are generated daily from a variety of medical devices and healthcare events. These medical data include structured data, semi-structured data, and unstructured data [68]. The experimental data are taken from the publicly available Brazilian ophthalmological dataset (BRSET) [69,70]. All data records are publicly available at the PhysioNet database. The images were directly obtained from the Nikon NF505 and Canon CR-2 devices in JPEG format without the application of any preprocessing techniques. All images were acquired with a focus on the macula and annotated by a retinal specialist ophthalmologist, following labeling criteria defined by the research team. All images were annotated by a retinal specialist ophthalmologist, following labeling criteria defined by the research team. The retinal labeling process was accompanied by metadata including the retinal imaging device used, patients’ nationality, age (in years), sex, clinical history, insulin usage, and duration of diabetes. Demographic and clinical details were derived from electronic medical records according to self-reported health information. The BRSET dataset includes 16,266 fundus images from 8,524 patients, with each image accompanied by demographic and clinical metadata. The aim is to validate the capability of the proposed multi-modal FKG-MM approach for handling large, variable and multi-modal datasets.

### Exploratory data analysis and preprocessing

**Fig 6:**
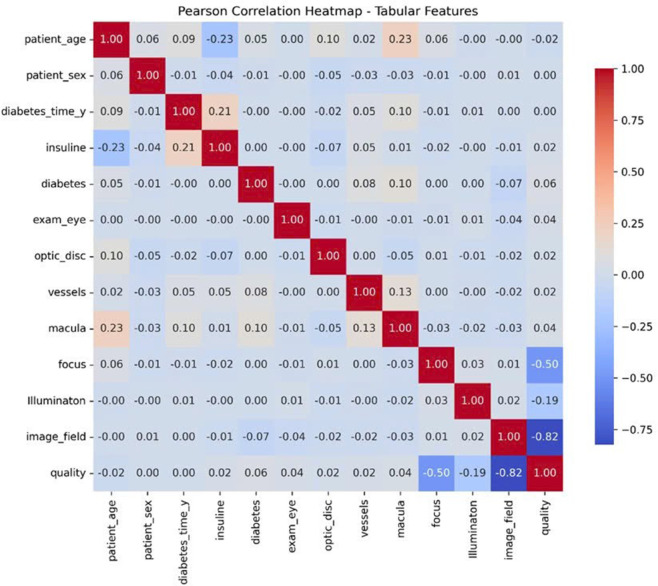
Heatmap tabular feature.

**Fig 7:**
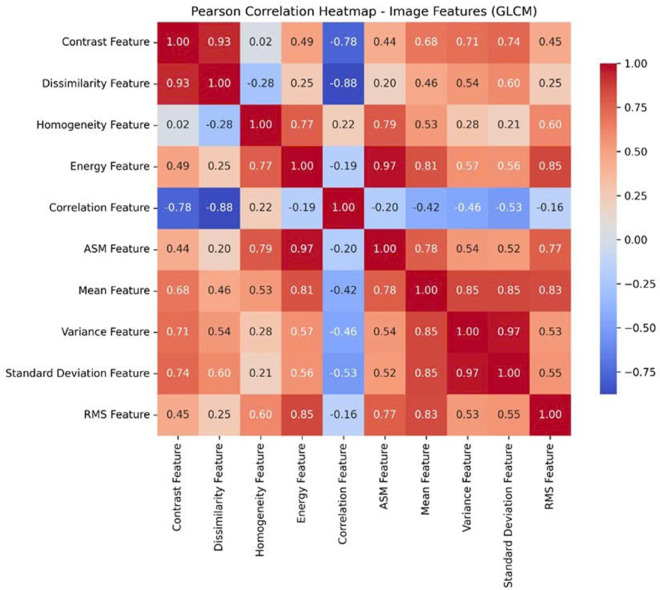
Heatmap image feature.

**Fig 8:**
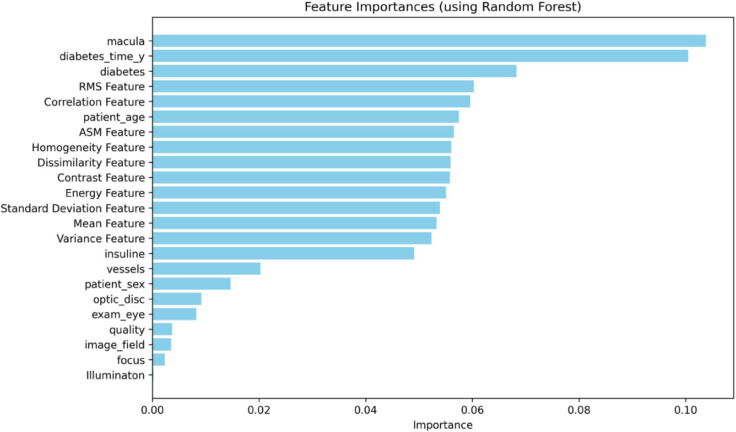
Feature importance chart.

Before training the model, data analysis and preprocessing play a particularly important role. The BRSET multi-modal dataset consists of images and tabular data. Correlation analysis of tabular data features is shown in the correlation matrix in Fig 6. With image data, after extracting GLCM features, features are evaluated for their correlation using the Heatmap chart in Fig 7. In addition, to evaluate the impact of both tabular and image-based data features on the output labels for diabetic retinopathy diagnosis, Feature importance was analyzed using the Random Forest algorithm. The analysis results, illustrated in Fig 8, indicate that the features macula, duration of diabetes (in years), and diabetes are the three most influential attributes. Notably, all three are derived from tabular data, underscoring the significant role of tabular information in determining the diagnostic outcomes for diabetic retinopathy.

### Experimental scenarios and evaluation metrics

In this study, for later convenience, the FKG model for unimodal data in [65] is called FKG-UM. To evaluate the effectiveness of the proposed method, experimental scenarios are conducted. During the experiment, the datasets were divided into a 70% training set and a 30% testing set.

The following scenarios are used to validate the performance of the proposed model:

(1) In the first scenario, fundus images are combined with medical records to obtain the FKG-MM. The evaluation criteria applied in this context include the accuracy and computational time. The aim of this scenario is to verify the hypothesis: “*FKG-MM is more accurate than FKG-UM but it is much more time-consuming overall*”.

(2) The second scenario is conducted to compare different fusion methods to find the best method among five feature fusions, including Feature Selection, Tensor Product Method, Hadamard Product Method, Filter Method, and Wrapper Method. The comparison criteria are the same as in scenario 1. In addition, ANOVA analysis was performed to assess the fluctuation in the experimental outcomes of Scenario 2 in light of the accuracy.

Considering the nature of the task, evaluation metrics such as precision, accuracy, and time-consuming for classification tasks were utilized to assess the effectiveness of the introduced model and mitigate overfitting. Detailed information about the evaluation metrics used is provided in Table 4, where:

- True Positive (TP) refers to a positive instance that is correctly classified as positive;
- True Negative (TN) denotes a negative instance that is accurately identified as negative;
- False Positive (FP) represents a negative instance that is incorrectly classified as positive;
- Negative (FN) corresponds to a positive instance that is incorrectly classified as negative.

**Table 4:** Performance evaluation metrics.

Metric	Formula	Description
Accuracy	$\frac{TP+FP}{TP+TN+FP+FN}$	Overall performance of the model
Time-consuming	Seconds	Total time for training and testing

The following sections will present the experimental results and evaluate the effectiveness of the proposed framework.

### The first scenario: Performance comparison of FKG-MM with FKG-UM

#### Experimental results.

Tables 5, 6, 7, and 8 are the results with different cases of the FKGS model when changing the sample rate and error threshold with the aim of evaluating the performance of the FKG-MM compared to the FKG-UM. With all tables above, in terms of accuracy, the FKG-MM model has significantly higher accuracy, achieving approximately 84-85% across all configurations, while the FKG-UM model has lower accuracy, ranging from 70-72%. Indeed, the improvement in accuracy when using both fundus images and medical records (FKG-MM) is approximately 12-14%. On the other hand, in terms of computational time, FKG-UM is a unimodal that always takes less time than that FKG-MM when integrating new data modality.

**Table 5:** Feature selection method with sampling ratio of 15% and error threshold of 0.2.

Model	Modality	Acc (%)	Training Time (s)	Testing Time (s)	Total Time (s)
**FKG-UM**	**Medical Records**	72.30	**44.75**	**429.05**	**473.79**
**FKG-MM**	**Fundus Image + Medical Records**	**84.19**	103.17	1461.34	1564.51

**Table 6:** Feature selection method with sampling ratio of 15% and error threshold of 0.3.

Model	Modality	Acc (%)	Training Time (s)	Testing Time (s)	Total Time (s)
**FKG-UM**	**Medical Records**	70.62	**44.50**	**430.06**	**474.56**
**FKG-MM**	**Fundus Image + Medical Records**	**84.00**	104.34	2139.62	2243.97

**Table 7:** Feature selection method with sampling ratio 20% and error threshold 0.2.

Model	Modality	Acc (%)	Training Time (s)	Testing Time (s)	Total Time (s)
**FKG-UM**	**Medical Records**	72.28	**87.62**	**585.97**	**673.59**
**FKG-MM**	**Fundus Image + Medical Records**	**85.35**	180.09	1626.59	1806.68

**Table 8:** Feature selection method with sampling ratio 20% and error threshold 0.3.

Model	Modality	Acc (%)	Training Time (s)	Testing Time (s)	Total Time (s)
**FKG-UM**	**Medical Records**	70.95	**85.23**	**577.58**	**662.81**
**FKG-MM**	**Fundus Image + Medical Records**	**85.22**	170.23	1460.32	1630.55

**Fig 9:**
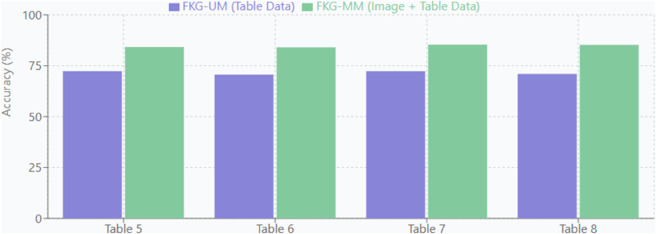
Comparison of accuracy.

The correlation of the accuracy of FKG-UM with FKG-MM is shown specifically and visually in Fig 9

#### ANOVA analysis.

To conduct ANOVA, the null hypothesis *H*_0_ is set up as follows:

**“*H***_**0**_ **: The accuracy of FKG-MM is significantly higher than that of FKG-UM, but is more time-consuming"**

As shown in the accuracy results in Table 9, it is observed that F=586.17, p<0.0001

**Table 9:** ANOVA results for accuracy.

Source of Variation	Sum of Squares (SS)	df	Mean Square (MS)	F	p-value
**Between Groups**	345.84	1	345.84	586.17	<0.0001
**Within Groups**	3.54	6	0.59		
**Total**	349.38	7			

Reject the null hypothesis *H*_0_: There is a significant difference in accuracy between FKG-MM and FKG-UM. FKG-MM has significantly higher accuracy than FKG-UM.

#### Main findings.

From the above results, some observations are given as follows:

- In environments requiring real-time processing or with limited computational resources, FKG-UM may be the appropriate choice.
- In applications demanding high accuracy where longer processing times are acceptable (such as offline analysis), FKG-MM would be a better option.
- If investment in more powerful hardware is possible, using FKG-MM will provide significant benefits in terms of accuracy.

### The second scenario: Finding the most effective multi-modal feature selection methods

#### Experimental results.

In scenario 2, experiments are conducted to evaluate the model performance on five feature selection methods when integrating multi-modal data to find the most effective method. To ensure objectivity, several parameter sets in the FKGS model are used to provide a basis for statistical analysis and ensure differentiation in the assessment. In the first parameter set, when the sampling rate is 15% and the error threshold is 0.2, the results are as shown in Table 10. The best performance in each column is shown in bold. The table shows that the Feature Selection method gives the highest accuracy while the Hadamard method gives the worst results. Also, in terms of time, the Wrapper method has the lowest total computation time and is much lower than the other methods.

**Table 10:** FKG-MM results with sampling ratio of 15% and error threshold of 0.2.

FKG-MM	Acc (%)	Training Time (s)	Testing Time (s)	Total Time (s)
**Feature Selection**	**84.19**	103.17	1461.34	1564.51
**Tensor**	81.18	125.47	1461.91	1587.38
**Hadamard**	77.01	58.25	1194.45	1252.71
**Filter**	81.40	98.65	1458.72	1557.38
**Wrapper**	77.22	**7.02**	**162.08**	**169.10**

**Fig 10:**
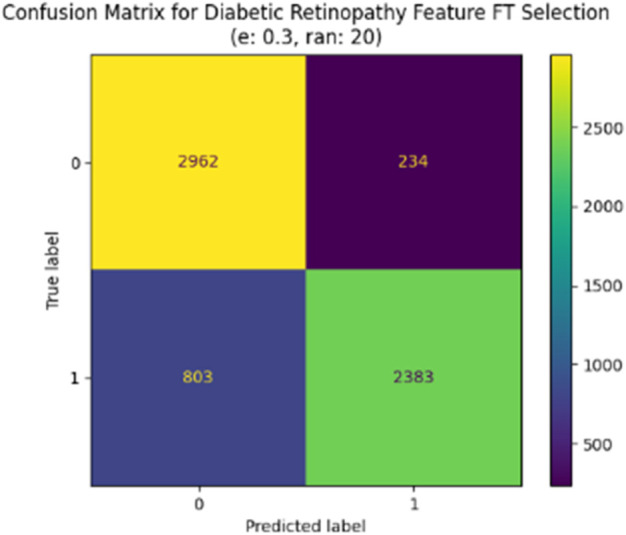
Confusion matrix of Feature Selection method with sample rate 20% and error threshold 0.3.

The same is true for the remaining parameter configurations. Specifically, Table 11 shows the experimental results with a sampling rate of 15%, and an error threshold of 0.3. The experimental results with a sample rate of 20% and an error threshold of 0.2 and 0.3 are shown in Tables 12 and 13, respectively. The confusion matrix for the best model is represented in Fig 10. As we are dealing with a binary classification problem, two classes can be seen in the matrix: 0 and 1. In this case, the diagonal elements represent correctly predicted true positives (TP) and true negatives (TN). The matrix does not exhibit any significant bias toward a particular class, indicating consistent predictions across all classes. By accurately classifying 85.22% of the test data, our proposed Feature Selection method demonstrates superior performance.

**Table 11:** FKG-MM results with sampling ratio of 15% and error threshold of 0.3.

FKG-MM	Acc (%)	Training Time (s)	Testing Time (s)	Total Time (s)
**Feature Selection**	**84.00**	104.34	2139.62	2243.97
**Tensor**	80.87	126.27	2156.41	2282.68
**Hadamard**	76.05	56.13	1886.40	1942.53
**Filter**	81.71	98.13	2140.51	2238.65
**Wrapper**	78.57	**7.19**	**160.20**	**167.38**

**Table 12:** FKG-MM results with sampling ratio of 20% and error threshold of 0.2.

FKG-MM	Acc (%)	Training Time (s)	Testing Time (s)	Total Time (s)
**Feature Selection**	**85.35**	180.09	1626.59	1806.68
**Tensor**	82.73	218.80	1625.48	1844.29
**Hadamard**	78.58	101.17	1402.12	1503.30
**Filter**	82.70	168.62	1633.02	1801.64
**Wrapper**	78.59	**13.05**	**217.36**	**230.42**

**Table 13:** FKG-MM results with sampling ratio of 20% and error threshold of 0.3.

FKG-MM	Acc (%)	Training Time (s)	Testing Time (s)	Total Time (s)
**Feature Selection**	**85.22**	170.23	1460.32	1630.55
**Tensor**	82.17	204.41	1413.33	1617.74
**Hadamard**	77.05	95.91	1276.62	1372.54
**Filter**	83.26	162.60	1466.17	1628.77
**Wrapper**	77.47	**13.42**	**214.80**	**228.23**

#### ANOVA analysis.

Now, ANOVA analysis of performance of 5 FKG-MM Methods is conducted to determine which method is the most effective.

- ANOVA results for accuracy: A significance level of 0.05 was selected, and the results of the ANOVA analysis are shown in Table 14. The analysis reveals a statistically significant difference in the total processing time across the five methods, with an F-value of 45.86 and a p-value of less than 0.0001. There is a statistically significant difference in total processing time among the 5 methods.
- ANOVA results for total computational time: As shown in Table 15, with an F-value of 28.17 and p <0.0001, there is a statistically significant difference in total processing time among the 5 methods.

**Table 14:** ANOVA results for accuracy.

Source of Variation	Sum of Squares (SS)	df	Mean Square (MS)	F	p-value
**Between Groups**	168.09	4	42.02	45.86	<0.0001
**Within Groups**	13.75	15	0.92		
**Total**	181.84	19			

**Table 15:** ANOVA results for total time.

Source of Variation	Sum of Squares (SS)	df	Mean Square (MS)	F	p-value
**Between Groups**	11,109,966	4	2,777,492	28.17	<0.0001
**Within Groups**	1,478,524	15	98,568		
**Total**	12,588,490	19			

#### Post-hoc test results.

To determine the difference of the methods from each other, the Tukey HSD test was conducted. The results of the posterior analysis for accuracy and the computational time are demonstrated in Tables 16 and 17. The results show that all pairs of methods have statistically significant differences in processing time.

**Table 16:** Tukey HSD results for accuracy.

Pair Comparison	Mean Difference	q-value	p-value	Conclusion
**Feature Selection - Filter**	2.42	5.04	<0.001	Significant difference
**Feature Selection - Tensor**	2.95	6.14	<0.001	Significant difference
**Feature Selection - Wrapper**	6.73	14.01	<0.001	Significant difference
**Feature Selection - Hadamard**	7.52	15.65	<0.001	Significant difference
**Filter - Tensor**	0.53	1.10	>0.05	Not significant
**Filter - Wrapper**	4.31	8.97	<0.001	Significant difference
**Filter - Hadamard**	5.10	10.62	<0.001	Significant difference
**Tensor - Wrapper**	3.78	7.87	<0.001	Significant difference
**Tensor - Hadamard**	4.57	9.51	<0.001	Significant difference
**Wrapper - Hadamard**	0.79	1.64	>0.05	Not significant

**Table 17:** Tukey HSD results for total time.

Pair Comparison	Mean Difference	q-value	p-value	Conclusion
**Feature Selection - Filter**	–40.20	0.26	0.9998	Not significan
**Feature Selection - Tensor**	66.61	0.42	0.9993	Not significant
**Feature Selection - Wrapper**	1567.63	9.99	<0.001	Significant difference
**Feature Selection - Hadamard**	248.64	1.58	0.8442	Not significant
**Filter - Tensor**	26.41	0.17	0.9999	Not significant
**Filter - Wrapper**	1607.83	10.24	<0.001	Significant difference
**Filter - Hadamard**	288.84	1.84	0.7910	Not significant
**Tensor - Wrapper**	1634.24	10.41	<0.001	Significant difference
**Tensor - Hadamard**	315.25	2.01	0.7486	Not significant
**Wrapper - Hadamard**	1318.99	8.40	<0.001	Significant difference

#### Main findings.

The main findings from the experiments are as follows:

- Feature Selection method has the highest accuracy across all four experimental conditions with an average of 84.69%.
- The Wrapper method has dramatically lower total execution times across all conditions, with an average of 198.78 seconds. This is approximately 7.6 times faster than the next fastest method (Hadamard).
- Overall ranking by accuracy: Feature Selection > Filter > Tensor > Wrapper > Hadamard
- Overall ranking by speed: Wrapper ≫ Hadamard > Filter > Feature Selection > Tensor

## Conclusion

In this study, a novel multi-modal data integration with fuzzy knowledge graph framework, which can be applied to various types of medical data was proposed. The proposed framework, FKG-MM, integrated data from different data sources and types. This is even more important and a step forward for clinician decision-making, which uses diverse information from multiple sources. From the experimental results, this study has contributed a multi-modal data integration model in image and tabular modality, applying some multi-modal data fusion methods and conducted experiments on a specific disease dataset in the healthcare domain. The results are very promising with significantly improved accuracy, while the processing performance is at an acceptable level.

However, this study also has some limitations. First, the study only uses at integrating 2 types of image and tabular data models without experimenting with other data modalities. In addition, available datasets are used without experiments being conducted on datasets from different sources with varying qualities. Furthermore, for diabetic retinopathy disease, this study only stops at the goal of diagnosing and detecting diseases without solving the problem of evaluating and classifying different levels of diseases. Finally, the proposed FKG-MM model is only in the initial testing phase, so that in order to be applied in clinical practice, it is necessary to carry out expert-based assessment and validation processes and select specific treatment regimens.

From the results achieved, this study can open up further research and development directions for effective application in clinical practice in the future. We will continue to improve the model to be able to integrate data from multiple sources and data from many different types of models such as 3D images, text, and videos to improve reliability and computational performance in supporting disease diagnosis, especially toward time-critical or resource-constrained settings.

In addition, in order for the framework to be put into practical applications, it is necessary to carry out standard procedures and external assessments, systematize the diagnostic process, and upgrade the data processing system to improve the quality of data from different sources. Specifically, it is recommended to implement diverse data-fusion strategies and integration mechanisms across many heterogeneous modalities to enhance the accuracy.

Regrding diabetic retinopathy, it is necessary to study and upgrade the model to be able to support diagnosis and classification of disease levels, and make decisions to support patient treatment at each specific disease stage. In general, the FKG-MM model shows promising potential to be applied to other domains such as smart education, smart transportation, digital banking, etc.

## Appendix

**A1 Source code and dataset.** The source code and dataset of this study can be found here:

https://github.com/thanhst/Fuzzy-Knowledge-Graph

https://drive.google.com/drive/folders/1L5NTkPrJgLF1Z-eds03iV5c0tDIH3s_D.

**Illustrative example 2** Below is the detailed calculation of example 2.

It is assumed that there is:

- An image feature matrix $F_{\text{img}}\in {\mathbb{R}}^{2\times 3}$ (2 samples, each with 3 features)
- A text feature matrix $F_{\text{tab}}\in {\mathbb{R}}^{2\times 2}$ (2 samples, each with 2 features)

$$F_{\text{img}}=[\begin{array}{ccc} 4 & 2 & 8\\ 6 & 1 & 3 \end{array}],\;F_{\text{tab}}=[\begin{array}{cc} 5 & 3\\ 9 & 2 \end{array}]$$

**Tensor Product Fusion: Step-by-Step**

**Step 1: Normalize Features.** First, each row vector is normalized using L2 normalization.

### For $F_{\text{img}}$:

Row 1: [4, 2, 8]


$$\text{Norm}=\sqrt{4^{2}+2^{2}+8^{2}}=\sqrt{84}\approx 9.165$$

Normalized:


$$\left[\frac{4}{9.165},\frac{2}{9.165},\frac{8}{9.165}\right]\approx [0.437,0.218,0.873]$$

Row 2: [6, 1, 3]


$$\text{Norm}=\sqrt{6^{2}+1^{2}+3^{2}}=\sqrt{46}\approx 6.782$$

Normalized:


$$\left[\frac{6}{6.782},\frac{1}{6.782},\frac{3}{6.782}\right]\approx [0.885,0.147,0.442]$$

Thus:


$${\text{normalizedF}}_{\text{img}}=[\begin{array}{ccc} 0.437 & 0.218 & 0.873\\ 0.885 & 0.147 & 0.442 \end{array}]$$

### For $F_{\text{tab}}$:

Row 1: [5, 3]


$$\text{Norm}=\sqrt{5^{2}+3^{2}}=\sqrt{34}\approx 5.831$$

Normalized:


$$\left[\frac{5}{5.831},\frac{3}{5.831}\right]\approx [0.858,0.515]$$

Row 2: [9, 2]


$$\text{Norm}=\sqrt{9^{2}+2^{2}}=\sqrt{85}\approx 9.220$$

Normalized:


$$\left[\frac{9}{9.220},\frac{2}{9.220}\right]\approx [0.976,0.217]$$

Thus:


$${\text{normalizedF}}_{\text{tab}}=[\begin{array}{cc} 0.858 & 0.515\\ 0.976 & 0.217 \end{array}]$$

**Step 2: Compute Tensor Product.** The tensor product of the normalized feature matrices is computed.

The unfolded tensor product (size 6×4) is computed:


$$\text{fullTensorProduct}=[\begin{array}{cccc} 0.375 & 0.225 & 0.426 & 0.095\\ 0.187 & 0.112 & 0.213 & 0.047\\ 0.749 & 0.450 & 0.852 & 0.189\\ 0.759 & 0.456 & 0.864 & 0.192\\ 0.118 & 0.076 & 0.144 & 0.032\\ 0.379 & 0.228 & 0.432 & 0.096 \end{array}]$$

**Step 3: Apply SVD Decomposition.** A truncated singular value decomposition (SVD) is applied:


$$\text{fullTensorProduct}\approx USV^{T}$$

Where:


$$U=[\begin{array}{cc} 0.351 & -0.218\\ 0.176 & -0.109\\ 0.702 & -0.436\\ 0.711 & 0.627\\ 0.118 & 0.104\\ 0.355 & 0.312 \end{array}],\;S=[\begin{array}{cc} 1.923 & 0\\ 0 & 0.283 \end{array}],\;V=[\begin{array}{cc} 0.856 & 0.127\\ 0.514 & 0.076\\ 0.975 & -0.095\\ 0.217 & -0.021 \end{array}]$$

**Step 4: Create Projection Matrices.** Taking the square root of *S*:


$$\sqrt{S}=[\begin{array}{cc} 1.387 & 0\\ 0 & 0.532 \end{array}]$$

Thus:


$$P_{\text{img}}=U\sqrt{S}=[\begin{array}{cc} 0.487 & -0.116\\ 0.244 & -0.058\\ 0.974 & -0.232\\ 0.986 & 0.334\\ 0.164 & 0.055\\ 0.492 & 0.166 \end{array}]\;P_{\text{tab}}=V\sqrt{S}=[\begin{array}{cc} 1.187 & 0.068\\ 0.713 & 0.040\\ 1.352 & -0.051\\ 0.301 & -0.011 \end{array}]$$

**Project Features and Concatenate.** The matrix $P_{\text{img}}$ is reshaped into two separate matrice:


$$P_{\text{img\_img1}}=[\begin{array}{cc} 0.487 & -0.116\\ 0.244 & -0.058\\ 0.974 & -0.232 \end{array}],\;P_{\text{img\_img2}}=[\begin{array}{cc} 0.986 & 0.334\\ 0.164 & 0.055\\ 0.492 & 0.166 \end{array}]$$

And $P_{\text{tab}}$ into:


$$P_{\text{tab\_tab1}}=[\begin{array}{cc} 1.187 & 0.068\\ 0.713 & 0.040 \end{array}],\;P_{\text{tab\_tab2}}=[\begin{array}{cc} 1.352 & -0.051\\ 0.301 & -0.011 \end{array}]$$

Projection results:


$$\text{imgProjection}={\text{normalizedF}}_{\text{img}}\times [P_{\text{img\_img1}};P_{\text{img\_img2}}]=[\begin{array}{cc} 1.157 & -0.276\\ 1.053 & 0.356 \end{array}]\;\text{tabProjection}={\text{normalizedF}}_{\text{tab}}\times [P_{\text{tab\_tab1}};P_{\text{tab\_tab2}}]=[\begin{array}{cc} 1.386 & 0.079\\ 1.384 & -0.052 \end{array}]$$

**Result.**


$$\text{fusedFeatures}=[\begin{array}{cccc} 1.157 & -0.276 & 1.386 & 0.079\\ 1.053 & 0.356 & 1.384 & -0.052 \end{array}]$$

The final fused feature matrix has dimensions [2×4], where:

- The first two columns contain the projected image features.
- The last two columns contain the projected text features.

**Illustrative example 3.** Below is the detailed calculation of example 3.

It is supposed that:

- Image feature matrix $F_{\text{img}}\in {\mathbb{R}}^{2\times 3}$ (2 samples, each with 3 features)
- Tabular feature matrix $F_{\text{tab}}\in {\mathbb{R}}^{2\times 2}$ (2 samples, each with 2 features)
- Common dimension: commonDim=2

Matrices are:


$$F_{\text{img}}=[\begin{array}{ccc} 1 & 2 & 3\\ 4 & 5 & 6 \end{array}]\;,\;F_{\text{tab}}=[\begin{array}{cc} 7 & 8\\ 9 & 10 \end{array}]$$

**Hadamard Product Fusion: Step-by-Step.**

**Step 0: Initialize Projection Matrices.** Initialize projection matrices:


$$W_{\text{img}}\leftarrow \text{InitializeMatrix}(p,\text{commonDim})\;W_{\text{tab}}\leftarrow \text{InitializeMatrix}(q,\text{commonDim})$$

Since $F_{\text{img}}$ has 3 features and $F_{\text{tab}}$ has 2 features, and A projection to a common dimension of 2 is desired:


$$W_{\text{img}}:3\times 2,\;W_{\text{tab}}:2\times 2$$

Assuming:


$$W_{\text{img}}=[\begin{array}{cc} 0.1 & 0.2\\ 0.3 & 0.4\\ 0.5 & 0.6 \end{array}],\;W_{\text{tab}}=[\begin{array}{cc} 0.7 & 0.8\\ 0.9 & 0.1 \end{array}]$$

**Step 1: Project Features.** Project features:


$$\text{imgProjected}\leftarrow F_{\text{img}}\times W_{\text{img}}\;\text{tabProjected}\leftarrow F_{\text{tab}}\times W_{\text{tab}}$$

Given:


$$F_{\text{img}}=[\begin{array}{ccc} 1 & 2 & 3\\ 4 & 5 & 6 \end{array}],\;F_{\text{tab}}=[\begin{array}{cc} 7 & 8\\ 9 & 10 \end{array}]$$

Calculate:


$$\text{imgProjected}=[\begin{array}{cc} 1\times 0.1+2\times 0.3+3\times 0.5 & 1\times 0.2+2\times 0.4+3\times 0.6\\ 4\times 0.1+5\times 0.3+6\times 0.5 & 4\times 0.2+5\times 0.4+6\times 0.6 \end{array}]=[\begin{array}{cc} 2.2 & 2.8\\ 4.9 & 6.4 \end{array}]$$

Similarly:


$$\text{tabProjected}=[\begin{array}{cc} 7\times 0.7+8\times 0.9 & 7\times 0.8+8\times 0.1\\ 9\times 0.7+10\times 0.9 & 9\times 0.8+10\times 0.1 \end{array}]=[\begin{array}{cc} 12.1 & 6.4\\ 15.3 & 8.2 \end{array}]$$

**Step 2: Normalize the Projected Features.** Normalize using L2 normalization:

For imgProjected:


$$\text{First row L2 norm}=\sqrt{2.2^{2}+2.8^{2}}=\sqrt{12.68}\approx 3.56\;\text{Second row L2 norm}=\sqrt{4.9^{2}+6.4^{2}}=\sqrt{64.97}\approx 8.06$$

Thus:


$$\text{imgNormalized}=[\begin{array}{cc} \frac{2.2}{3.56} & \frac{2.8}{3.56}\\ \frac{4.9}{8.06} & \frac{6.4}{8.06} \end{array}]=[\begin{array}{cc} 0.618 & 0.787\\ 0.608 & 0.794 \end{array}]$$

For tabProjected:


$$\text{First row L2 norm}=\sqrt{12.1^{2}+6.4^{2}}=\sqrt{187.37}\approx 13.69\;\text{Second row L2 norm}=\sqrt{15.3^{2}+8.2^{2}}=\sqrt{301.33}\approx 17.36$$

Thus:


$$\text{tabNormalized}=[\begin{array}{cc} \frac{12.1}{13.69} & \frac{6.4}{13.69}\\ \frac{15.3}{17.36} & \frac{8.2}{17.36} \end{array}]=[\begin{array}{cc} 0.884 & 0.468\\ 0.881 & 0.472 \end{array}]$$

**Step 3: Compute the Hadamard Product.** Compute element-wise (Hadamard) product:


$$\text{hadamardProduct}=\text{imgNormalized}⊙\text{tabNormalized}\;=[\begin{array}{cc} 0.618\times 0.884 & 0.787\times 0.468\\ 0.608\times 0.881 & 0.794\times 0.472 \end{array}]=[\begin{array}{cc} 0.546 & 0.368\\ 0.536 & 0.375 \end{array}]$$

**Step 4: Apply Non-linear Transformation.** Apply ReLU activation (no change because all elements are positive):


$$\text{activatedFeatures}=[\begin{array}{cc} 0.546 & 0.368\\ 0.536 & 0.375 \end{array}]$$

Concatenate the activated features, normalized image, and normalized tabular features:


$$\text{fusedFeatures}=[\begin{array}{cccccc} 0.546 & 0.368 & 0.618 & 0.787 & 0.884 & 0.468\\ 0.536 & 0.375 & 0.608 & 0.794 & 0.881 & 0.472 \end{array}]$$

Project to the common dimension using a projection matrix:


$$W_{\text{fusion}}=[\begin{array}{cc} 0.1 & 0.2\\ 0.3 & 0.4\\ 0.5 & 0.6\\ 0.7 & 0.8\\ 0.9 & 0.1\\ 0.2 & 0.3 \end{array}]$$

Thus:


$$\text{Final fusedFeatures}=\text{fusedFeatures}\times W_{\text{fusion}}$$

Calculating:


First row:0.055+0.110+0.309+0.551+0.796+0.094=1.9150.109+0.147+0.371+0.630+0.088+0.140=1.485Second row:0.054+0.113+0.304+0.556+0.793+0.094=1.9140.107+0.150+0.365+0.635+0.088+0.142=1.487

Thus:


$$\text{Final fusedFeatures}=[\begin{array}{cc} 1.915 & 1.485\\ 1.914 & 1.487 \end{array}]$$

**Result.** The final output of the Hadamard Product Fusion algorithm is:


$$\text{fusedFeatures}=[\begin{array}{cc} 1.915 & 1.485\\ 1.914 & 1.487 \end{array}]$$

This fused representation combines information from both image and tabular features, capturing the interactions between corresponding dimensions through the Hadamard product.

**Illustrative example 4.** Below is the detailed calculation of example 4.

Given:


$$F_{\text{img}}=[\begin{array}{ccccc} 5 & 2 & 8 & 6 & 9\\ 7 & 1 & 4 & 3 & 8\\ 6 & 3 & 9 & 5 & 7 \end{array}]\;\text{(5 image features)}\;F_{\text{tab}}=[\begin{array}{cccc} 3 & 6 & 2 & 7\\ 8 & 5 & 1 & 9\\ 4 & 7 & 3 & 8 \end{array}]\;\text{(4 tabular features)}\;\text{target}=[\begin{array}{ccc} 0 & 1 & 0 \end{array}]\;\text{(Target variable)}$$

Other parameters:


$$k_{\text{img}}=2\;\text{(Number of image features to select)}\;k_{\text{tab}}=2\;\text{(Number of tabular features to select)}$$

**Filter-based multi-modal Feature Selection: Step-by-Step Solution.** I’ll solve this example of the Filter-based multi-modal Feature Selection algorithm step-by-step with the given data.

**Input Data Analysis.**

- *F*_*img*_: Image feature matrix (3×5) with 5 features.
- *F*_*tab*_: Tabular feature matrix (3×4) with 4 features.
- target=[0,1,0]: Target variable.
- *k*_*img*_ = 2: Need to select 2 image features.
- *k*_*tab*_ = 2: Need to select 2 tabular features.

**Step 1: Calculate Importance Scores for Each Feature.** Using two criteria, mutual information and random forest importance.

**Mutual Information (MI) between each feature and the target:**

- For image features:
  $$\text{MI}(img_{1},\text{target})=0.38\;\text{MI}(img_{2},\text{target})=0.21\;\text{MI}(img_{3},\text{target})=0.52\;\text{MI}(img_{4},\text{target})=0.27\;\text{MI}(img_{5},\text{target})=0.35$$
- For tabular features:
  $$\text{MI}(tab_{1},\text{target})=0.31\;\text{MI}(tab_{2},\text{target})=0.43\;\text{MI}(tab_{3},\text{target})=0.22\;\text{MI}(tab_{4},\text{target})=0.40$$

**Random forest importance:**

- For image features:
  $$\text{RF}(img_{1},\text{target})=0.18\;\text{RF}(img_{2},\text{target})=0.11$$
  $$\text{RF}(img_{3},\text{target})=0.35\;\text{RF}(img_{4},\text{target})=0.15\;\text{RF}(img_{5},\text{target})=0.21$$
- For tabular features:
  $$\text{RF}(tab_{1},\text{target})=0.22\;\text{RF}(tab_{2},\text{target})=0.32\;\text{RF}(tab_{3},\text{target})=0.13\;\text{RF}(tab_{4},\text{target})=0.33$$

**Combine scores (average):**

- For image features:
  $$\text{Score}(img_{1})=\frac{0.38+0.18}{2}=0.28\;\text{Score}(img_{2})=\frac{0.21+0.11}{2}=0.16\;\text{Score}(img_{3})=\frac{0.52+0.35}{2}=0.435\;\text{Score}(img_{4})=\frac{0.27+0.15}{2}=0.21\;\text{Score}(img_{5})=\frac{0.35+0.21}{2}=0.28$$
- For tabular features:
  $$\text{Score}(tab_{1})=\frac{0.31+0.22}{2}=0.265\;\text{Score}(tab_{2})=\frac{0.43+0.32}{2}=0.375\;\text{Score}(tab_{3})=\frac{0.22+0.13}{2}=0.175\;\text{Score}(tab_{4})=\frac{0.40+0.33}{2}=0.365$$

**Step 2: Rank Features by Importance Scores.** Image features (from highest to lowest):

- *img*_3_: 0.435
- *img*_1_: 0.28
- *img*_5_: 0.28
- *img*_4_: 0.21
- *img*_2_: 0.16

**Tabular features (from highest to lowest):**

- *tab*_2_: 0.375
- *tab*_4_: 0.365
- *tab*_1_: 0.265
- *tab*_3_: 0.175

**Step 3: Select a Larger Initial Set of Candidate Features.**

- Select 3 image features (more than *k*_*img*_): *img*_3_, *img*_1_, *img*_5_
- Select 3 tabular features (more than *k*_*tab*_): *tab*_2_, *tab*_4_, *tab*_1_

**Step 4: Remove Highly Correlated Features Within Each Modality.** Correlation matrix among selected image features:


$$\text{Corr}(img_{3},img_{1})=0.15\;\text{Corr}(img_{3},img_{5})=0.78\;\text{(high)}\;\text{Corr}(img_{1},img_{5})=0.22$$

Since $\text{Corr}(img_{3},img_{5})$ is high, and *img*_3_ has a higher score than *img*_5_, we remove *img*_5_.

Remaining image features: *img*_3_, *img*_1_

Correlation matrix among selected tabular features:


$$\text{Corr}(tab_{2},tab_{4})=0.31\;\text{Corr}(tab_{2},tab_{1})=0.25\;\text{Corr}(tab_{4},tab_{1})=0.63\;\text{(high)}$$

Since $\text{Corr}(tab_{4},tab_{1})$ is high, and *tab*_4_ has a higher score than *tab*_1_, we remove *tab*_1_.

Remaining tabular features: *tab*_2_, *tab*_4_

**Step 5: Analyze Cross-Modal Correlation.** Correlation between selected image and tabular features:


$$\text{Corr}(img_{3},tab_{2})=0.23\;\text{Corr}(img_{3},tab_{4})=0.17\;\text{Corr}(img_{1},tab_{2})=0.21\;\text{Corr}(img_{1},tab_{4})=0.29$$

All cross-modal correlations are low, indicating that the selected features provide complementary information.

**Step 6: Combine the Selected Features.** The final selected feature set is:


$$F_{fused}=[img_{3},img_{1},tab_{2},tab_{4}]$$

**Final Result.** The algorithm has selected:

- 2 image features: *img*_3_ and *img*_1_
- 2 tabular features: *tab*_2_ and *tab*_4_

This is the optimal feature set based on importance criteria, with low intra-modal correlation and complementary information across modalities.

**Illustrative example 5.** Below is the detailed calculation of example 5.

Let me provide a step-by-step example illustrating the **Wrapper-based multi-modal Feature Selection** algorithm.

The following data will be utilized:

- *F*_*img*_: Image feature matrix with features [img_1, img_2, img_3, img_4, img_5]
- *F*_*tab*_: Tabular feature matrix with features [tab_1, tab_2, tab_3, tab_4]
- target: Target variable
- max_img = 3 (Maximum image features to select)
- max_tab = 2 (Maximum tabular features to select)
- min_img_features = 1 (Minimum image features required)
- min_tab_features = 1 (Minimum tabular features required)

**Step 1: Define evaluation model and performance metric.**

- Model: Random Forest classifier
- Metric: F1-score

**Step 2: Initialize empty feature sets.**

- selected_img_indices = []
- selected_tab_indices = []
- best_score = −∞

**Step 3: Pre-filter features to reduce search space.** After applying a simple filter method, we keep:

- Image features: [img_1, img_2, img_3, img_5]
- Tabular features: [tab_1, tab_2, tab_4]

**Step 4: Ensure minimum representation from each modality.** First, select the minimum required features from each modality:

**For image features** (min_img_features = 1):

- F1(img_1) = 0.65
- F1(img_2) = 0.58
- F1(img_3) = 0.72
- F1(img_5) = 0.61

img_3 gives the highest score, so selected_img_indices = [3].

**For tabular features** (min_tab_features = 1):

- F1(tab_1) = 0.56
- F1(tab_2) = 0.69
- F1(tab_4) = 0.64

tab_2 gives the highest score, so selected_tab_indices = [2].

**Step 5 & 6: Perform Sequential Forward Selection.**

Starting with selected_img_indices = [3] and selected_tab_indices = [2].

Current best_score = 0.69 (from tab_2).

**Iteration 1**:

- For remaining image features:
  - F1([img_3, img_1] + [tab_2]) = 0.76
  - F1([img_3, img_2] + [tab_2]) = 0.71
  - F1([img_3, img_5] + [tab_2]) = 0.73
- For remaining tabular features:
  - F1([img_3] + [tab_2, tab_1]) = 0.72
  - F1([img_3] + [tab_2, tab_4]) = 0.78

The best new score is 0.78 from adding tab_4. Update:

- selected_tab_indices = [2, 4]
- best_score = 0.78

**Iteration 2**:

- For remaining image features:
  - F1([img_3, img_1] + [tab_2, tab_4]) = 0.83
  - F1([img_3, img_2] + [tab_2, tab_4]) = 0.77
  - F1([img_3, img_5] + [tab_2, tab_4]) = 0.80
- For remaining tabular features:
  - F1([img_3] + [tab_2, tab_4, tab_1]) = 0.79

The best new score is 0.83 from adding img_1. Update:

- selected_img_indices = [3, 1]
- best_score = 0.83

**Iteration 3**:

- For remaining image features:
  - F1([img_3, img_1, img_2] + [tab_2, tab_4]) = 0.82
  - F1([img_3, img_1, img_5] + [tab_2, tab_4]) = 0.85
- For remaining tabular features:
  - F1([img_3, img_1] + [tab_2, tab_4, tab_1]) = 0.84

The best new score is 0.85 from adding img_5. Update:

- selected_img_indices = [3, 1, 5]
- best_score = 0.85

**Iteration 4**:

- We’ve reached max_img = 3 for image features.
- For remaining tabular features:
  - F1([img_3, img_1, img_5] + [tab_2, tab_4, tab_1]) = 0.84

No improvement, so the algorithm is stopped.

**Step 7: Combine the selected features.**

Final selected features:

- Image features: img_3, img_1, img_5
- Tabular features: tab_2, tab_4

*F*_*fused*_ is formed by concatenating these selected features.

The final feature set is [img_3, img_1, img_5, tab_2, tab_4] with a performance score of 0.85.

This example demonstrates how the wrapper-based approach systematically evaluates combinations of features from both modalities to find the optimal subset that maximizes the model’s performance on the specific task.
